# Causal AI for cancer immunotherapy: a narrative framework review of target trial emulation, treatment-effect learning and clinical translation

**DOI:** 10.3389/fimmu.2026.1896755

**Published:** 2026-08-20

**Authors:** Xin Zhao, Zhuoran Liu, Jie Yang, Qingyun Xie, Tianyang Mao, Heng Zhou, Hongyuan Wang, Peng Zheng, Kangyi Jiang, Fengwei Gao

**Affiliations:** 1 Department of Hepato-Pancreato-Biliary Surgery, People's Hospital of Leshan, Leshan, China; 2 Laboratory of Hepatic AI Translation, Frontiers Science Center for Disease-Related Molecular Network, West China Hospital, Sichuan University, Chengdu, China; 3 Department of General Surgery, West China Hospital, Sichuan University, Chengdu, China; 4 Liver Transplant Center, Transplant Center, West China Hospital, Sichuan University, Chengdu, China

**Keywords:** cancer immunotherapy, causal AI, clinical translation, heterogeneous treatment effects, immune checkpoint inhibitors, multimodal artificial intelligence, target trial emulation, treatment-effect learning

## Abstract

This narrative framework Review examines how artificial intelligence (AI) can move cancer immunotherapy research from outcome prediction toward target-trial-based treatment-effect learning. AI has produced increasingly accurate models for predicting response, survival and immune-related toxicity during cancer immunotherapy. Yet most models estimate outcome risk under observed care rather than the causal effect of choosing one strategy over another. We therefore frame immunotherapy AI as a causal digital-medicine problem: clinically useful AI should begin with a target-trial question that specifies eligibility, time zero, treatment strategies, comparators, outcomes, estimands and bias-control plans before model development. Within this framework, multimodal AI outputs from imaging, digital pathology, omics, microbiome data, electronic health records and clinical text can function as baseline confounders, candidate effect modifiers, longitudinal state variables or outcome-ascertainment tools. We distinguish established causal-inference approaches, such as target trial emulation, propensity-score weighting, g-methods, TMLE, DML and heterogeneous-treatment-effect estimation, from exploratory technologies such as reinforcement learning and digital twins that require prospective safety validation. We close by outlining validation, reporting, workflow, regulatory and lifecycle-monitoring requirements for moving from predictive biomarkers to trustworthy causal learning systems in immuno-oncology.

## 1 Introduction: the decision gap in AI-enabled cancer immunotherapy

Immune checkpoint inhibitors (ICIs) have changed the therapeutic logic of solid tumours. By blocking inhibitory immune pathways, including CTLA-4, PD-1 and PD-L1, ICIs can produce durable disease control in a subset of patients and have moved immunotherapy from selected tumour types into a central role across cancers, lines of therapy and treatment strategies (1, 2). Benefit, however, is far from universal. Many patients do not achieve objective responses or long-term survival benefit, and some experience immune-related adverse events, pseudoprogression, hyperprogression, acquired resistance or relapse after treatment discontinuation. The central question in immuno-oncology has therefore shifted from whether immunotherapy can work to which patients are most likely to derive net benefit, at what time, in which combinations, for what duration and at what level of risk.

This shift has made clinical decision-making substantially more complex. Single biomarkers such as PD-L1 expression, tumour mutational burden, microsatellite instability and mismatch repair deficiency have provided important tools for patient selection, but their predictive value varies by tumour type, assay platform, threshold, tumour heterogeneity, line of therapy and combination regimen. They cannot, in isolation, explain the full spectrum of immunotherapy response. Recent reviews and multi-omics studies suggest that efficacy and toxicity arise from interactions among tumour-intrinsic genomic features, antigen presentation, interferon signalling, T-cell infiltration, myeloid suppression, stromal barriers, metabolic remodelling, the gut microbiome and prior treatment pressure (3–9). Decision-oriented immunotherapy models therefore need to integrate imaging, pathology, molecular omics, microbiome data, circulating biomarkers, electronic health records and longitudinal follow-up rather than rely on a single static marker.

Artificial intelligence (AI) has entered immuno-oncology in response to this complexity. Machine-learning and deep-learning models can extract phenotypic patterns from high-dimensional, multimodal and nonlinear data that are difficult to capture with conventional statistical models. These models have been used to predict ICI response, progression-free survival, overall survival, pathological response, surrogate biomarkers such as PD-L1 or MSI, and the risk of immune-related toxicity. Digital pathology and imaging AI can infer histological or radiological proxy phenotypes of the tumour immune microenvironment from routine clinical data. Multi-omics and single-cell AI can link mechanisms of immune escape to cellular states. Large language models and multimodal foundation models offer new ways to integrate medical records, literature, trial protocols and molecular reports (10–14). Together, these developments position AI as an important interface between complex biological data and immunotherapy stratification.

Most existing studies, however, remain within a prediction paradigm. Models are usually trained on labels such as response, progression, survival or toxicity and are assessed with metrics such as the area under the receiver operating characteristic curve, C-index, accuracy, sensitivity, specificity or calibration. Such models ask whether a particular outcome is likely to occur. Clinical strategy selection asks a different question. What would happen if the same patient received ICI monotherapy rather than chemoimmunotherapy? What would be the long-term benefit and toxicity risk if surgery were advanced after neoadjuvant treatment, if immunotherapy were extended or stopped, or if the patient were rechallenged? A model may accurately identify a patient with poor prognosis without showing that the patient has greater relative benefit from one treatment. Similarly, a model may predict a high risk of immune-related adverse events without determining whether the expected net benefit is lower than that of an alternative strategy.

**Figure 1:**
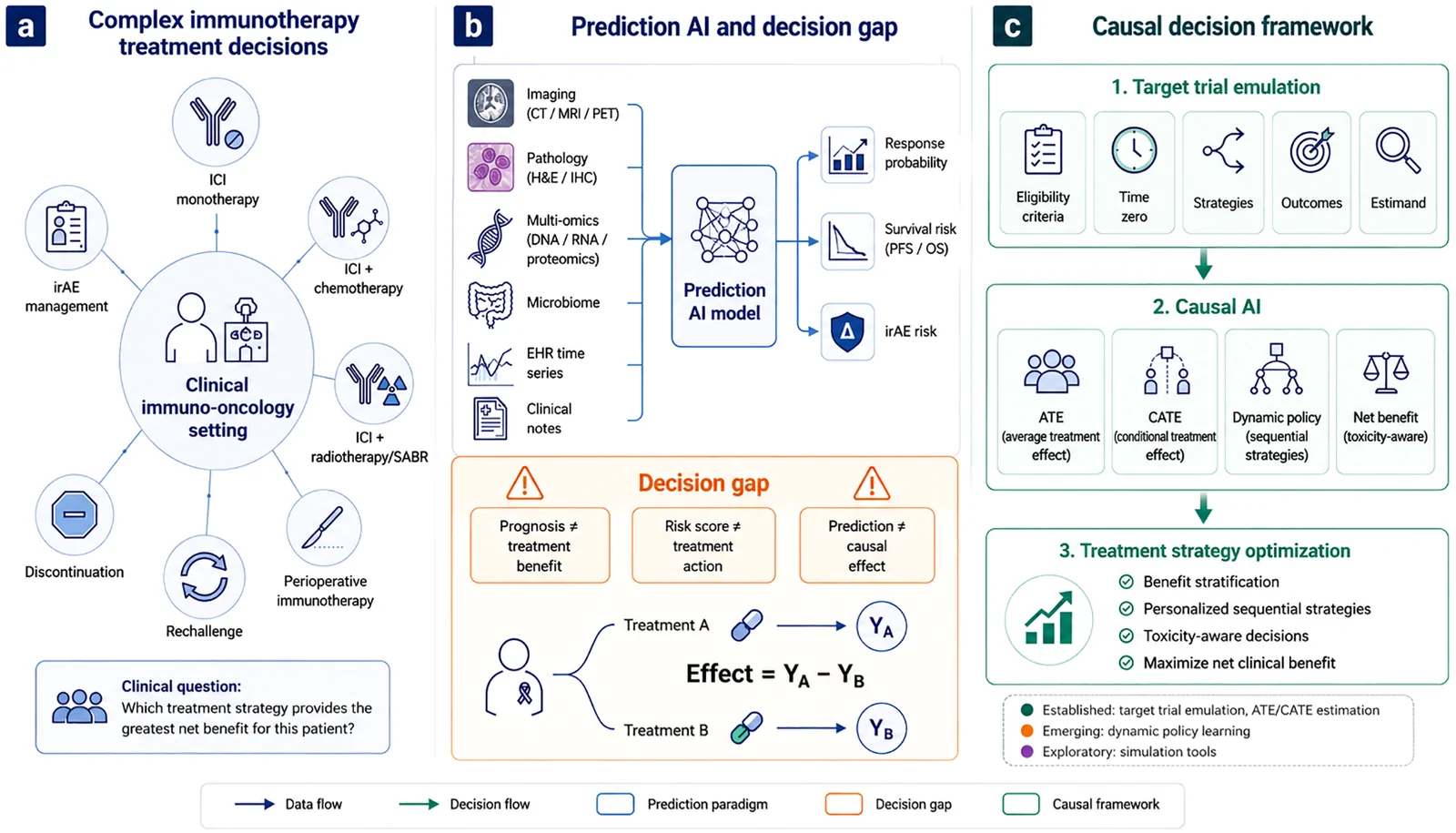
Causal digital-medicine roadmap for cancer immunotherapy. **(A)** Contemporary immuno-oncology decisions include treatment intensification, radiotherapy/stereotactic ablative radiotherapy (SABR), perioperative therapy, discontinuation, rechallenge and immune-related adverse event (irAE) management. **(B)** Prediction AI integrates imaging, pathology, multi-omics, microbiome, electronic health record (EHR) time series and clinical text to estimate response, survival and toxicity risks. These outputs are useful measurement layers, but require an explicit decision contrast before they can support treatment selection. **(C)** The causal framework begins with target trial emulation--eligibility criteria, time zero, strategies, outcomes and estimand--and then applies causal AI to estimate average and conditional treatment effects, dynamic policies and toxicity-aware net benefit. The maturity legend separates established design/estimation components from emerging dynamic-policy learning and exploratory simulation tools.

AI studies in immunotherapy therefore face a question-formulation problem as much as a performance problem. Prognostic models estimate outcome risk under observed care, whereas strategy optimization requires explicit comparison of feasible interventions, including average effects, heterogeneous effects, time-varying effects and toxicity-aware net benefit. In this narrative framework Review, causal AI refers to machine-learning-assisted causal inference and decision modelling under explicit target-trial designs, not to algorithms that infer causality automatically. Recent broad-scope reviews of AI in HLA immunogenomics, oncology clinical trials and real-time cancer care show that AI is reshaping biological discovery, patient matching, monitoring and clinical workflow (15–17). Our review narrows this broad landscape to a specific translational question: how can multimodal AI phenotypes be embedded in target-trial emulation and treatment-effect learning to support immunotherapy strategy decisions? Section 2 recasts imaging, pathology, omics, microbiome and text-derived AI outputs as causal modelling inputs; Sections 3–5 define the causal design and methods; and Sections 6–8 connect them to clinical use cases, validation and learning-system governance. This roadmap is summarized in **Figure 1**. **Tables 1**–**3** provide, respectively, a simplified overview of multimodal AI phenotypes as causal inputs, an example target trial emulation, and a target trial emulation checklist for AI-enabled immunotherapy studies.

**Table 1:** Simplified overview of multimodal AI phenotypes as causal inputs in cancer immunotherapy studies.

Data modality	Representative AI phenotype	Primary causal role	Common misuse to avoid	Translation priority
Imaging/radiomics	Baseline tumour burden, spatial heterogeneity and longitudinal response features from CT, MRI or PET/CT	Baseline confounder, effect modifier or longitudinal state depending on time zero	Using post-treatment response or pneumonitis as a baseline covariate	Protocol harmonization, external validation and leakage checks
Digital pathology	Spatial immune context, MSI/dMMR, TIL/TLS and tissue-architecture phenotypes from H&E, IHC or multiplex images	Mechanistic effect modifier or enrichment variable	Treating a slide-derived risk score as evidence of treatment benefit without a comparator	Cross-centre validation for scanner, staining and site effects
Omics/single-cell/spatial	Genomic, transcriptomic, scRNA-seq and spatial-proteomic immune or resistance states	Effect-modifier discovery and mechanistic priors	Using high-dimensional biomarkers as standalone causal evidence	Predefined hypotheses, shrinkage and independent validation
Microbiome/metabolomics	Microbial genes, metabolites, antibiotics and medication-linked host-immune signals	Modifiable exposure, confounder or effect modifier within a TTE	Inferring drug or microbiome effects without controlling diet, geography and indication	Repeated sampling and sensitivity analyses for confounding
EHR/time series	ECOG, laboratory values, medications, treatment-line history, follow-up and irAE trajectories	Core TTE data source and dynamic-regime state	Relying on uncurated administrative proxies or conditioning on post-treatment events	Line-of-therapy validation, missing-data modelling and censoring checks
LLMs/foundation models	Structured variables extracted from notes, reports, protocols and literature with traceable retrieval	Measurement and workflow support, not causal effect estimation	Using LLM output as a causal estimator or unverified endpoint	Traceable RAG, audit logs, human oversight and version control

This simplified table highlights the main causal role and most important misuse to avoid for each AI modality. The same AI phenotype may be a baseline covariate, effect modifier, mediator, time-varying confounder or outcome-ascertainment tool depending on the target-trial time zero and estimand. Post-treatment variables should be used only in prespecified longitudinal, mediation or landmark analyses, not as baseline covariates. AI, artificial intelligence; dMMR, mismatch repair deficiency; EHR, electronic health record; IHC, immunohistochemistry; irAE, immune-related adverse event; LLM, large language model; MSI, microsatellite instability; RAG, retrieval-augmented generation; TIL, tumour-infiltrating lymphocyte; TLS, tertiary lymphoid structure; TTE, target trial emulation.

**Table 2:** Example target trial emulation: first-line ICI monotherapy versus chemoimmunotherapy in metastatic NSCLC.

Component	Operational specification	Target estimand/ statistical notation
Eligibility	Adults with treatment-naive metastatic NSCLC at the first-line systemic-treatment decision point; no contraindication to either strategy; actionable EGFR, ALK or other driver-positive disease excluded or analysed separately; PD-L1 stratum prespecified, such as PD-L1 >=50% when monotherapy is guideline-eligible; baseline ECOG, tumour burden, organ function and comorbidity data available before time zero.	Target population: P = {E = 1, L0 observed before t0}.
Time zero	Date of first-line treatment decision or initiation, aligned across both strategies. Patients should not be required to survive until first imaging assessment or complete a fixed number of cycles before cohort entry.	Index date t0. H0 = L0 measured before t0.
Strategies	Strategy A: guideline-eligible PD-1 or PD-L1 inhibitor monotherapy within a prespecified molecular and PD-L1 stratum. Strategy B: platinum-based chemoimmunotherapy. Regimen, cycle number, maintenance, local therapy, switching and discontinuation rules should be defined before analysis.	Baseline A: A = 0, ICI monotherapy; A = 1, chemoimmunotherapy. g0/g1 executable.
Estimand and contrast	Intention-to-treat effect of initiating chemoimmunotherapy versus ICI monotherapy; secondary per-protocol or as-treated effects may be assessed with explicit censoring and weighting rules.	Primary ITT: Psi_ITT(tau) = E[Y1 - Y0 | E = 1]. Survival: Delta_RMST(tau) = E[min(T1,tau) - min(T0,tau) | E = 1]. Per-protocol/as-treated: clone-censor-weight or IPCW.
Outcomes	Overall survival, progression-free survival, restricted mean survival time, grade 3 or higher irAEs, treatment discontinuation, hospitalization and toxicity-weighted net benefit.	Y_E(tau), Y_T(tau), utility U. U = w_E benefit - w_T toxicity - w_B burden. Weights prespecified.
AI-enabled variables	Baseline imaging and pathology features for tumour burden and immune context; omics and microbiome features as candidate effect modifiers; NLP-derived treatment-line, toxicity and outcome labels; on-treatment imaging or ctDNA only in sequential landmark trials.	Use X_AI in L0 only if measured before t0. On-treatment imaging/ctDNA enters Lk in landmark or sequential trials, not baseline models.
Bias and sensitivity checks	Balance diagnostics, positivity assessment, missing-data handling, inverse-probability-of-censoring weights, negative-control or quantitative bias analysis where possible, external validation and subgroup transportability checks.	Checks: exchangeability; consistency; positivity; independent censoring after IPCW; overlap, missingness and sensitivity analyses.

**Table 3:** Target trial emulation checklist for AI-enabled immunotherapy studies.

Target trial component	Key question	Immunotherapy-specific examples	Common mistakes/biases	AI-enabled opportunities
Eligibility criteria	Who is the target population, and are patients at the same clinical decision point?	First-line advanced NSCLC; adding ICI after SABR in early inoperable NSCLC; perioperative immunotherapy; discontinuation after deep response (56–59)	Using post-treatment information for eligibility; including only patients who completed cycles or had follow-up imaging, causing selection bias	EHR, imaging and pathology phenotyping; identification of treatment lines, tumour states and comparable populations; trial enrichment (63)
Time zero	What is the shared start point at which all eligible strategies are possible?	ICI initiation date; decision after neoadjuvant therapy to proceed to surgery or extend treatment; landmark time after CR/PR for discontinuation	Defining time zero at first response assessment or another post-treatment event, creating immortal time bias (57)	Automatic extraction of index dates, treatment start/stop dates and scan dates; detection of time-zero misalignment
Treatment strategies	Which executable and reproducible strategies are being compared?	ICI monotherapy vs chemoimmunotherapy; SABR vs SABR plus ICI; continuing ICI vs planned discontinuation; rechallenge vs switching after irAE	Vague exposure definitions; *post hoc* classification of treatment paths; ignoring treatment switching, discontinuation and cross-over	NLP and structured-data extraction of regimens, dose, cycles and combination/sequencing; strategy encoding
Assignment procedure	How can random assignment be emulated and confounding controlled?	Adjust for ECOG, tumour burden, PD-L1/MSI/TMB, organ function, prior therapy, comorbidities and drug access	Confounding by indication; unmeasured key variables; positivity violations; high-risk patients receiving only one strategy	High-dimensional propensity scores, DML nuisance models, missing-data modelling, balance diagnostics and subgroup positivity checks
Follow-up	When does follow-up start and end, and how are censoring and switching handled?	6-month pCR/MPR; 12- or 24-month EFS/PFS; OS; irAE windows; relapse follow-up after discontinuation	Differential follow-up; counting survival to response assessment as treatment benefit; informative censoring	Longitudinal EHR and imaging extraction; IPCW, MSMs and dynamic landmark updates; missingness and follow-up pattern detection
Outcomes	How are efficacy, toxicity and net benefit defined?	OS, PFS, RMST, pCR/MPR, grade 3 or higher irAEs, treatment discontinuation, quality of life and composite net benefit	Focusing on a single efficacy endpoint while ignoring irAEs, quality of life, discontinuation and future treatment opportunities	Multi-outcome models, joint efficacy-toxicity utility, patient-preference weighting and text-based irAE extraction (65)
Causal contrast/estimand	Is the target effect ITT, per-protocol, as-treated or a dynamic strategy effect?	Effect of initiating chemoimmunotherapy; effect of adhering to a 2-year strategy; strategy effect after irAE interruption or rechallenge	Unclear estimand; interpreting risk prediction as treatment benefit; not specifying handling of deviations from the strategy	Align AI risk/effect stratification with ATE, ATT, CATE, RMST differences or dynamic policy outputs
Sensitivity and reporting	How will unmeasured confounding, measurement error, transportability and transparency be addressed?	Multicentre real-world TTE; rare or weak-evidence populations; cross-platform imaging/pathology models	Reporting only the main model; not disclosing the target-trial protocol and real-world data mapping; poor reproducibility	TARGET checklist, bias checks, external validation, drift monitoring and automated reporting support (60–62)

This checklist is intended for observational or hybrid studies that use AI-enabled data extraction, phenotyping or covariate construction to emulate an immunotherapy target trial. The central requirement is to define the hypothetical randomized trial before analysing the data and then document how each trial component is mapped to real-world records. AI may support implementation but does not replace specification of eligibility, time zero, treatment strategies, outcomes, estimands or bias checks. AI, artificial intelligence; CATE, conditional average treatment effect; CR, complete response; EHR, electronic health record; ICI, immune checkpoint inhibitor; IPCW, inverse probability of censoring weighting; irAE, immune-related adverse event; MSM, marginal structural model; PR, partial response; RMST, restricted mean survival time; TMB, tumour mutational burden; TTE, target trial emulation.

## 2 Review methodology and evidence scope

This article is a narrative framework review rather than a systematic review or meta-analysis. We searched PubMed, Web of Science, Google Scholar and reference lists for English-language methodological papers, clinical studies, systematic reviews, guidelines and reporting standards relevant to cancer immunotherapy, target trial emulation, causal inference, heterogeneous treatment effects, multimodal AI, LLM-assisted evidence extraction, reinforcement learning, digital twins and clinical AI translation through June 2026. Search concepts included cancer immunotherapy, immune checkpoint inhibitor, target trial emulation, causal machine learning, treatment-effect heterogeneity, digital pathology, radiomics, multi-omics, microbiome, electronic health records, large language models, reinforcement learning, digital twins and clinical decision support. We prioritized peer-reviewed publications, randomized or target-trial-like treatment comparisons, externally validated AI studies, and established causal-inference methodology. Preprints, meeting abstracts and simulation-only studies were not used as stand-alone clinical evidence; when mentioned, they were labelled as exploratory or hypothesis-generating.

Terminology was standardized throughout the manuscript. Causal AI denotes AI-assisted causal-inference and decision-modelling workflows under an explicit estimand. Causal machine learning denotes estimators such as TMLE, DML, causal forests, meta-learners and survival causal forests that can use flexible nuisance or effect-modification models. Treatment-effect learning denotes estimation of ATE, ATT, CATE, RMST differences, dynamic treatment-regime effects or toxicity-weighted utility. Decision AI denotes the clinical workflow layer that communicates model outputs to multidisciplinary teams. None of these terms implies that an algorithm can infer causality without a prespecified target trial, identification assumptions and validation.

## 3 Multimodal AI phenotypes as inputs to causal immunotherapy studies

**Figure 2:**
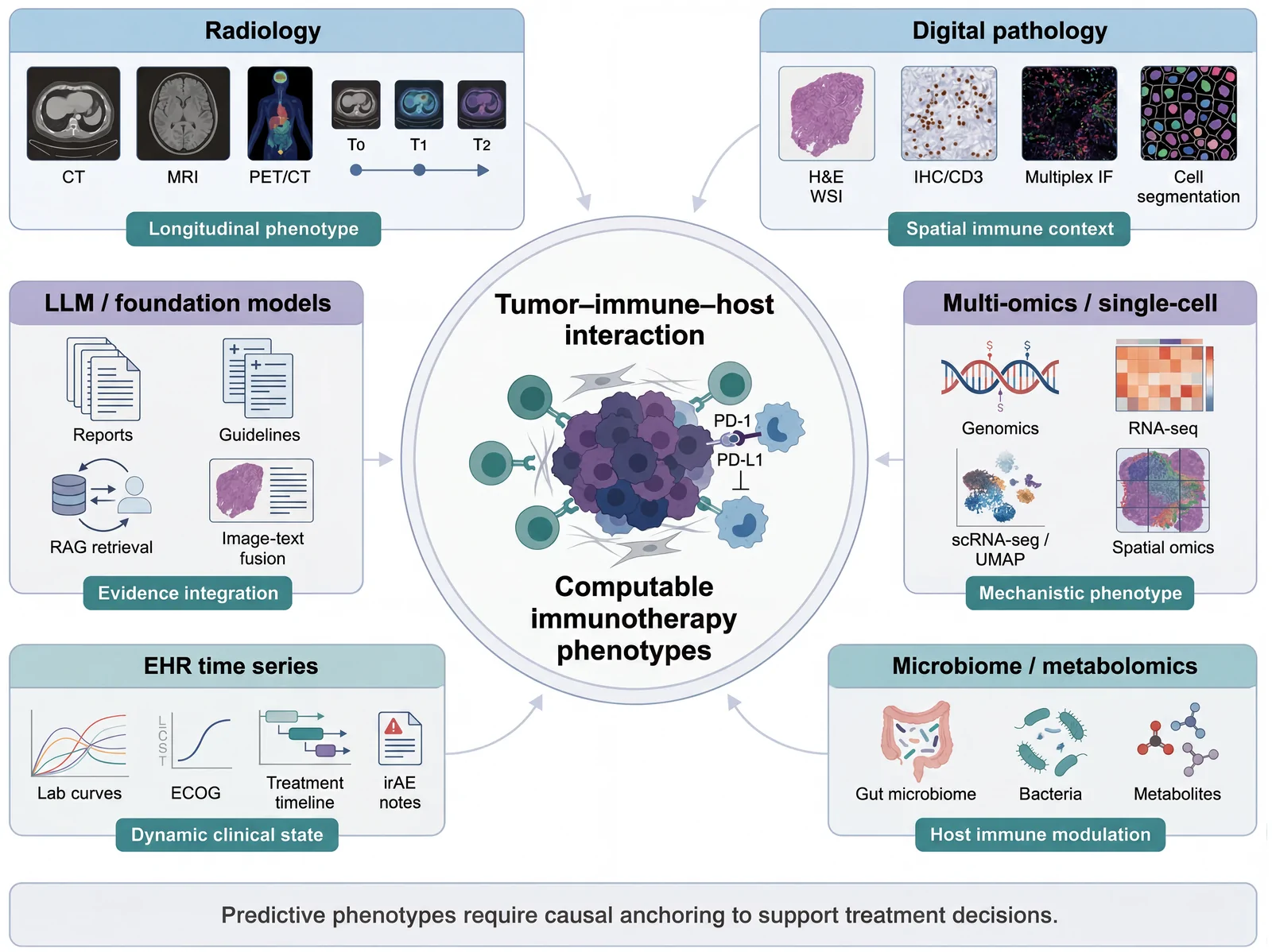
Multimodal AI phenotypes and their causal roles in immunotherapy strategy studies. Radiology can quantify tumour burden, spatial heterogeneity and longitudinal response; digital pathology can characterize tissue architecture and spatial immune context; multi-omics, single-cell and spatial profiling can resolve mechanistic immune states; microbiome and metabolomics can describe host immune modulation; EHR time series can represent dynamic clinical states; and LLM or foundation-model workflows can support evidence integration and outcome extraction. Each phenotype must be anchored to a specified treatment decision, time zero and estimand before being used as a baseline confounder, effect modifier, longitudinal state variable, mediator or outcome-ascertainment tool.

The central question for AI in immunotherapy is not simply which model best predicts response, but which AI-derived measurements can support a credible comparison of feasible treatment strategies. Current AI systems generate computable phenotypes from radiology, digital pathology, molecular omics, single-cell and spatial profiling, microbiome data, EHR time series and clinical text. Outside a causal design, these phenotypes usually remain risk scores or surrogate biomarkers. Within a target trial, however, their roles can be specified: pretreatment imaging and pathology may capture baseline disease burden and immune context; omics and microbiome features may identify candidate effect modifiers; on-treatment ctDNA, imaging change and irAEs may become longitudinal state variables; and LLM-extracted text can improve treatment-line, toxicity and outcome ascertainment. Section 2 therefore summarizes AI modalities according to their causal role rather than as isolated prediction tasks. The resulting modality-to-causal-role mapping is summarized in **Figure 2**.

### 3.1 Imaging AI as tumour burden, effect modifier, and longitudinal state

Medical imaging is one of the most mature and accessible modalities for AI in immunotherapy. CT, MRI and PET/CT can be acquired before, during and after treatment, making them well suited to quantify tumour burden, metabolic activity, necrosis, vascularity, spatial heterogeneity and longitudinal change. CT-based radiomics and deep-learning signatures have been used to predict PD-L1 expression non-invasively and to assess immunotherapy outcomes in non-small cell lung cancer (NSCLC). Serial CT deep-learning models can use pretreatment and follow-up imaging to predict survival in patients with NSCLC treated with immunotherapy, suggesting that longitudinal imaging may capture early immune-treatment dynamics better than a single RECIST measurement (18, 19). In hepatocellular carcinoma (HCC) and perioperative settings, systematic reviews of radiomics-based machine-learning models for ICI response report generally favourable discrimination, but also highlight heterogeneity between studies, small sample sizes and limited external validation. Studies of neoadjuvant immunotherapy in NSCLC and multiparametric MRI combined with deep learning and radiomics in HCC have further extended imaging AI toward prediction of pathological response and progression-free survival benefit (20–22).

Imaging AI is also moving into perioperative and organ-preservation contexts. In neoadjuvant immunotherapy for NSCLC, machine-learning models have mainly aimed to predict major or complete pathological response, but systematic reviews have noted limited data scale, opaque handling of missing data, insufficient external validation and a high risk of bias in many studies (21). PET/CT radiomics has been used to infer PD-L1 expression from metabolic heterogeneity, offering a complementary non-invasive assessment of immune status (23). In thoracic neoadjuvant treatment, interpretable multimodal radiopathomics may help estimate pathological response and the feasibility of organ preservation, although clinical deployment requires solutions for scan protocols, image reconstruction, region-of-interest annotation, cross-centre transportability and workflow integration (24). Thus, the value of imaging AI lies not in replacing a single radiological readout but in providing computable inputs for treatment intensification, de-escalation, timing of surgery and organ-preserving strategies.

### 3.2 Digital pathology as spatial immune context and candidate effect modifier

Digital pathology is another central pillar of AI-enabled immuno-oncology. Routine H&E whole-slide images encode tumour architecture, necrosis, stroma, lymphocytic infiltration, tertiary lymphoid structures and spatial immune configurations. Deep learning has achieved clinical-grade MSI detection from H&E slides in colorectal cancer, indicating that routine pathology images can provide a low-cost, high-throughput entry point for molecular screening relevant to immunotherapy (25). AI-based digital pathology has since expanded from MSI and mismatch repair deficiency detection to PD-L1, tumour mutational burden, tumour-infiltrating lymphocytes, immune phenotypes, treatment response and survival. Models based on H&E, CD3 staining or multiplex immunohistochemistry can quantify immune infiltration, identify tumour-immune spatial relationships and provide prognostic or multi-outcome predictions in colon cancer, melanoma and multicentre colorectal cancer cohorts (26–30).

The clinical importance of digital pathology is that it may change the form of companion diagnostics. Conventional companion diagnostics often depend on a single immunohistochemical or molecular threshold, whereas computational pathology can integrate cell location, tissue architecture, staining intensity and spatial context into continuous risk or response scores (29). Yet labels for digital pathology AI are often derived from retrospective outcomes or weak slide-level annotations. Models may learn scanner, staining, institutional or sample-selection differences rather than true immunotherapy sensitivity. Digital pathology AI is therefore an important input layer for causal decision AI, but it cannot replace explicit comparison of treatment strategies.

### 3.3 Multi-omics, single-cell, and microbiome features as mechanistic causal inputs

Multi-omics, single-cell and microbiome data bring AI closer to the mechanistic basis of immunotherapy response. Multimodal deep-learning models have fused gene expression and pathology images to predict immunotherapy-relevant phenotypes in gastric adenocarcinoma. Machine-learning models based on single-cell RNA sequencing can identify ICI-response-associated genes and cellular states while preserving cellular resolution. Integrated analyses of tumour and immune-cell transcriptomes have further implicated B-cell and immune-interaction signals in primary resistance in metastatic melanoma (31–34). In lung adenocarcinoma, the integration of bulk and single-cell transcriptomic data has also been used to construct risk models based on metabolism-related epithelial-cell features, connecting tumour metabolism, immune landscape and prognosis (35).

Gut microbiome and circulating biomarkers add host-level and systemic dimensions to immunotherapy AI. Pan-cancer gene-level gut microbiome signatures have been proposed as predictors of ICI response, suggesting that biological priors and functional microbial features may improve cross-cohort generalization (33). Reviews of multimodal biomarkers in NSCLC similarly indicate that circulating tumour DNA can reflect molecular residual disease and on-treatment tumour burden, the microbiome can capture host immune regulation, and radiomics can summarize spatial heterogeneity and macroscopic phenotype. Integration across these domains is likely to outperform a single-biomarker strategy (36). The next generation of immunotherapy AI will therefore depend on cross-scale integration, spanning cellular states, tissue architecture, tumour genomics, host ecology and changes from baseline to on-treatment states.

### 3.4 Transformers, foundation models, and LLMs as representation and workflow tools

As data sources expand from structured variables to pathology images, clinical notes, molecular reports, trial protocols and guideline text, transformers, foundation models and large language models (LLMs) are increasingly being applied in immuno-oncology. Pretrained clinical transformers can use self-supervised learning and transfer learning to improve prediction of treatment efficacy and biomarkers in small clinical datasets. Vision-language foundation models align pathology images with text reports and show promise in pathology benchmarks, molecular marker prediction and NSCLC immunotherapy-response prediction (37, 38). LLMs have mainly been used for immuno-oncology question answering, patient education, evidence extraction, literature screening and virtual tumour boards. Comparative studies suggest that LLM responses to immuno-oncology questions can be readable, but may be inaccurate or incomplete; virtual tumour boards and multidisciplinary decision tools are better viewed as expert-augmentation systems than as substitutes for clinical accountability (39–42).

In this Review, LLMs are proposed primarily for evidence extraction, literature screening, protocol drafting, variable harmonization, treatment-line annotation and toxicity or outcome ascertainment. They should not be interpreted as causal effect estimators. Any treatment-effect estimate must still arise from a target-trial protocol, valid outcome and treatment definitions, measured confounding control, positivity assessment and sensitivity analysis.

### 3.5 Synthesis: causal roles depend on time zero and estimand

Current AI research in immunotherapy has therefore produced a broad measurement infrastructure, but the same variable can have different causal meanings depending on the decision point. Pretreatment radiomics, pathology and omics features may be legitimate baseline covariates in a first-line treatment trial. The same features measured after treatment initiation may be mediators, response markers or time-varying confounders, and should not be handled as ordinary baseline predictors. Treating a mediator, such as early ctDNA decline, tumour shrinkage, steroid initiation or an irAE, as if it were a baseline feature can block part of the treatment effect, condition on a treatment-affected collider, import endpoint information into the feature set and create post-treatment bias. LLM-derived irAE labels or treatment-line annotations can strengthen real-world outcome ascertainment, but they do not themselves estimate treatment effects. The central task is to align each AI phenotype with a target-trial time zero, treatment strategy and estimand. Once this alignment is explicit, multimodal AI becomes a causal measurement layer rather than a collection of independent prediction models.

## 4 Why prediction is not enough for treatment strategy selection

The preceding section shows that AI can extract rich computable phenotypes from imaging, pathology, multi-omics, microbiome and clinical text. Richer phenotyping, however, does not automatically produce better treatment decisions. Most models ask whether an outcome will occur; treatment selection asks whether the outcome would change under a different strategy. This distinction determines study design, variable selection, model interpretation and clinical translation.

### 4.1 From outcome prediction to counterfactual treatment questions

Prediction models estimate outcome probability under the observed data-generating process. They can support risk communication and follow-up planning, but treatment decisions require comparison of potential outcomes under alternative strategies, such as ICI monotherapy, chemoimmunotherapy, radiotherapy-immunotherapy, treatment extension, planned discontinuation or rechallenge. In the framework of Hernan and Robins, causal questions require explicit treatment, comparator, time zero, follow-up, outcome and target effect rather than retrospective interpretation of observed associations (43, 44).

This distinction is particularly important in immunotherapy because efficacy, toxicity and treatment adaptation are time dependent. Treatment choice is shaped by performance status, tumour burden, molecular test results, prior therapy, comorbidities, clinician preference and access. A high-performing prognostic model may mainly learn which patients are healthier or more likely to receive intensification, not who benefits more from one strategy. AI for immunotherapy treatment selection must estimate the expected incremental benefit of changing strategy, not merely the risk of a future outcome (45–47).

### 4.2 Prognosis, predictive biomarkers, and treatment benefit are not interchangeable

In clinical practice, poor prognosis, a high probability of response and large treatment benefit are often conflated, but they are not methodologically equivalent. A prognostic model estimates outcome risk under the current care pathway. A predictive biomarker identifies patients more likely to respond to a given treatment. Treatment benefit requires comparison between treatment and control. Kent and colleagues argued that the goal of personalized medicine is not simply to improve risk prediction but to estimate heterogeneous treatment effects (HTEs) more reliably, while avoiding the low power, multiplicity and interpretive problems of conventional single-variable subgroup analyses (45). This distinction applies directly to immunotherapy: high PD-L1 expression, high tumour mutational burden or a high-risk imaging phenotype may indicate response probability or natural prognosis, but do not necessarily imply larger net benefit relative to an alternative treatment.

A recent analysis of individual patient data from 10 randomized trials of atezolizumab illustrates this point. The investigators developed a machine-learning pretreatment prognosis score and tested whether it modified ICI efficacy. Except for IMvigor211, most trials supporting atezolizumab indications did not show a stable and significant interaction between baseline prognostic risk and treatment efficacy (48). Thus, even a risk model that separates high-risk from low-risk patients well cannot automatically be used as evidence of ICI benefit. Clinically, treatment-effect heterogeneity models are needed; prognostic scores should not be repurposed as treatment-selection tools without causal evidence.

### 4.3 Why high AUC is not a decision rule

Several recent immunotherapy studies have begun to move from risk prediction toward treatment-effect-oriented AI. Examples include individual treatment-effect estimation based on the I-SABR randomized trial, potential-outcomes comparisons of single-arm trials with historical controls, mutation-treatment interactions in real-world clinico-genomic data, strategy selection between ICI monotherapy and chemoimmunotherapy in metastatic NSCLC, and counterfactual tissue-generation models for studying the immune microenvironment (49–53). These studies suggest that AI outputs can shift from who will respond to which strategy is more likely to add benefit, but this shift requires a strict separation between predictive performance, model explanation and causal effect estimation.

AUC, C-index and accuracy measure discrimination, not the clinical net benefit of a treatment strategy. A model may have a high AUC but be poorly calibrated near a decision threshold. It may accurately identify patients who are likely to progress without showing whether progression risk can be reduced by a particular intervention. Immunotherapy decisions also involve toxicity, cost, quality of life and downstream treatment opportunities; a single risk score rarely maps directly onto an action. In HCC, a study comparing Cox regression with LLMs for long-term progression prediction found that structured Cox models outperformed several LLMs at 12, 24 and 36 months, underscoring that model complexity alone does not guarantee better clinical prediction or decision support (54). For immunotherapy AI, this is a cautionary example: if the question remains a risk-prediction problem, more complex models may improve apparent fit without addressing treatment effect or actionability.

Explainable AI studies further show that standard performance metrics can hide flawed model behaviour. HIPPO and related counterfactual explanation frameworks for computational pathology systematically modify tissue regions in whole-slide images to generate image-level counterfactuals, testing whether predictions rely on histologically plausible evidence and revealing model limitations that may not be visible from conventional metrics or attention maps (55). In immunotherapy, such counterfactual explanations are not equivalent to causal treatment-effect estimation. They nevertheless reinforce a shared principle: models should be tested against clinically and biologically plausible changes in inputs or actions, not judged only by offline accuracy.

### 4.4 Emerging examples of treatment-effect oriented AI in immunotherapy

Although most immunotherapy AI remains predictive, several studies have started to estimate treatment effects. I-SABR-SELECT is a representative example. Using the I-SABR randomized trial in early-stage inoperable NSCLC and the external STARS cohort, the study combined clinical and radiomic variables with counterfactual reasoning to estimate the individual treatment effect of adding immunotherapy to SABR. The model not only predicted outcomes but also generated treatment-switch recommendations. Patients treated according to model recommendations had better event-free survival, and among patients recommended for SABR plus immunotherapy, the restricted mean survival time benefit of adding immunotherapy was larger (49). The key contribution was not radiomics per se, but the expression of model output as a strategy-effect difference rather than as response prediction under a single treatment path.

Potential-outcomes analyses comparing single-arm trials with historical controls also illustrate how treatment effects may be estimated when randomization is difficult. In a single-arm study of adjuvant nivolumab for locally recurrent head and neck squamous-cell carcinoma, investigators used the Rubin causal model to compare 39 trial participants with untreated historical controls and estimated the average treatment effect on 2-year disease-free survival using Cox models, random survival forests and Bayesian additive regression trees. Despite substantial uncertainty, all three methods supported nivolumab over control (50). Such work shows how causal inference can help single-arm oncology studies move from observing favourable outcomes to estimating effects relative to an explicit comparator. Its credibility, however, depends on comparable controls, adequate covariates, assumptions about unmeasured confounding and sensitivity analyses; it should not be treated as a substitute for randomized evidence.

Large real-world clinico-genomic datasets are also being used to identify treatment-effect modifiers. A study of 78,287 patients across 20 cancer types systematically characterized mutation-treatment interactions and then developed an immunotherapy response risk score in advanced NSCLC (51). Another study addressed a clinically closer question in metastatic NSCLC: how to choose between ICI monotherapy and chemoimmunotherapy. Using real-world clinico-genomic data and weighted subgroup algorithms, the investigators built an ensemble model to identify patient subgroups more likely to benefit from treatment intensification (52). These studies are important proof-of-concept examples, but their clinical interpretation depends on treatment availability, measured confounding, adequate overlap, endpoint consistency and external transportability. Subgroup algorithms may discover clinically plausible benefit strata, yet such strata should be treated as candidates for prospective validation unless calibration, decision-curve performance, uncertainty and treatment-effect stability are demonstrated across centres and treatment eras.

### 4.5 Counterfactual representations as a bridge, not a substitute for causal design

Counterfactual generative models provide another tool for exploring mechanisms in the immune microenvironment. CF-HistoGAN uses highly multiplexed tissue imaging to generate artificial counterfactual tissue samples that remain as close as possible to the original tissue while expressing features of another outcome group. This approach can increase sensitivity for detecting differences in protein expression and spatial organization between outcome groups (53). It is well suited to hypothesis generation, for example asking which cells or protein channels would need to change if a non-responder-like tissue state were shifted toward a responder-like state.

Generative counterfactuals, however, should not be conflated with causal treatment effects. Image- or tissue-level counterfactual samples are typically generated in a statistical representation space and are used to explain models or propose mechanistic hypotheses. Treatment-effect estimation requires an explicitly intervenable strategy, temporal ordering, control of confounding and identification assumptions. These methods should therefore be viewed as bridges from phenotype discovery to causal design, not as causal inference in themselves. Without a target-trial question, even biologically plausible counterfactual images cannot answer whether a patient should receive additional immunotherapy.

### 4.6 Implications for the next stage: from prediction models to target trials

The next stage of immunotherapy AI is not simply to build larger models, fuse more data or maximize AUC. It is to redefine the research question before model development: who is eligible, what is time zero, which executable strategies are compared, how switching or discontinuation will be handled, which outcomes and follow-up windows matter, whether the target is average or heterogeneous benefit, and what action threshold is clinically acceptable. Once these elements are explicit, multimodal AI can support covariate construction, effect-modifier discovery, propensity or outcome modelling, dynamic updating and individualized strategy optimization.

## 5 Target trial emulation as a design framework for immunotherapy research

Target trial emulation (TTE) provides the design framework for comparing immunotherapy strategies in real-world data. It asks investigators to specify the protocol of an ideal randomized trial and then map its eligibility criteria, treatment assignment, follow-up and analysis to observational data as closely as possible. For immunotherapy, this design step is critical because ICI benefit may be delayed, and treatment selection is influenced by tumour burden, performance status, PD-L1, MSI or tumour mutational burden testing, prior therapy, comorbidities and drug access. If time zero, treatment strategies and outcome windows are not defined at the outset, sophisticated AI may amplify design bias rather than correct an ill-posed causal question.

### 5.1 From a predictive question to a trial-like causal question

**Figure 3:**
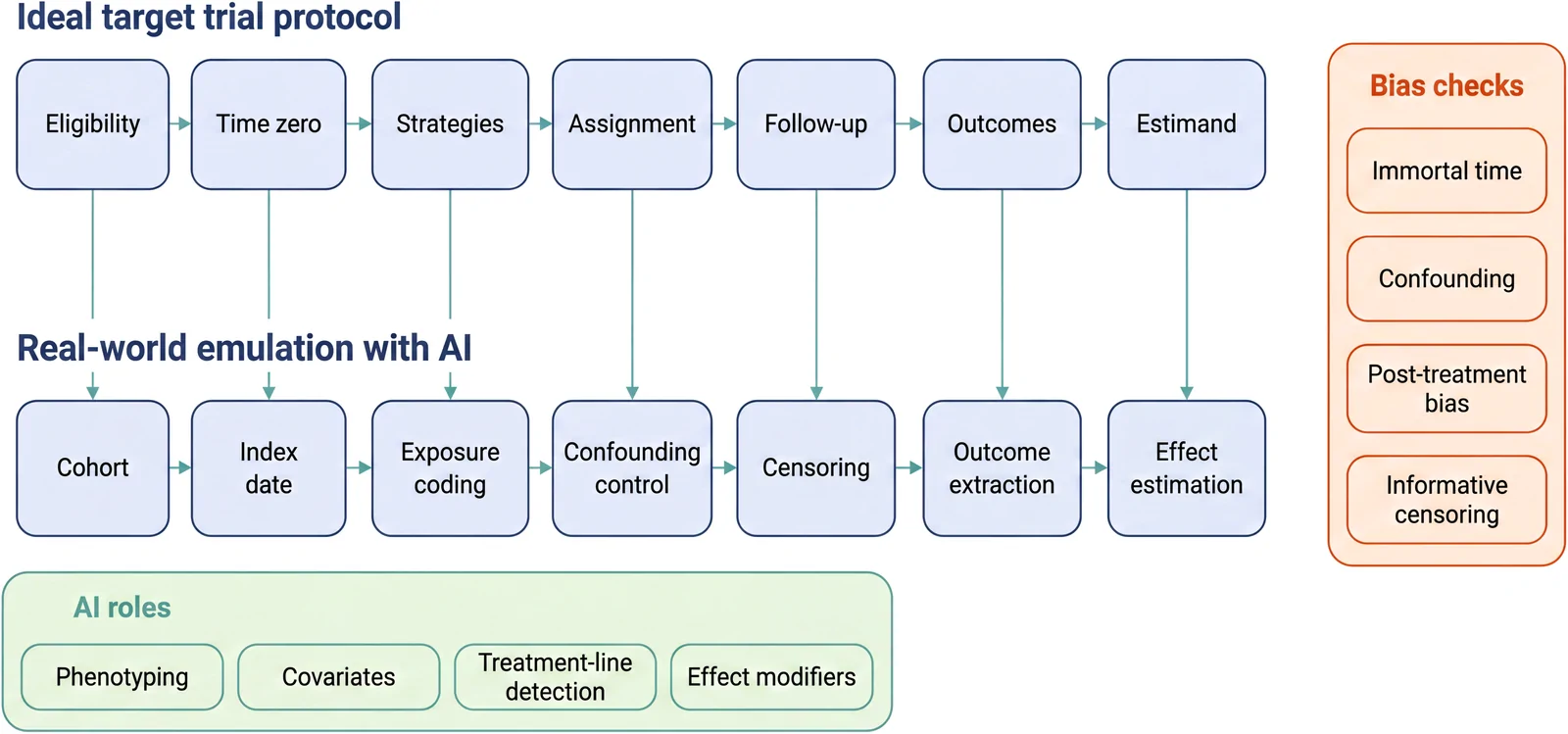
Target trial emulation workflow for AI-enabled immunotherapy studies. An ideal target trial first specifies eligibility, time zero, treatment strategies, assignment, follow-up, outcomes and estimand. Real-world emulation then maps these components to cohort construction, index-date definition, exposure coding, confounding control, censoring, outcome extraction and effect estimation. AI can improve phenotyping, covariate ascertainment, treatment-line detection and effect-modifier discovery, but the design must explicitly address immortal-time, confounding, post-treatment and informative-censoring biases.

The first step in TTE is not modelling but translation of a clinical question into a treatment comparison that could, in principle, be randomized. The target-trial protocol should define eligibility, treatment strategies, assignment procedure, start of follow-up, outcomes, causal contrast and analysis plan (56). Alignment of eligibility, treatment assignment and follow-up start is essential to avoid immortal-time and related design biases (57, 58). TTE clarifies the question and reduces self-inflicted design bias, but it cannot solve missing data, measurement error, unmeasured confounding or poor representativeness (59). Within this protocol, AI-derived imaging, pathology, omics and clinical-text phenotypes have assignable temporal and causal roles as baseline covariates, effect modifiers, stratification variables or dynamic state variables. The corresponding real-world emulation workflow is shown in **Figure 3**.

### 5.2 Preventing design biases before modelling begins

A second value of TTE is that it exposes common biases before modelling begins. These biases are frequent in real-world immunotherapy studies. If eligibility requires completion of at least two ICI cycles and follow-up imaging, patients who die early or discontinue early will be excluded, making ICI appear more beneficial. If time zero is defined at the first response assessment rather than at the treatment decision, the survival time required to reach assessment will be incorrectly counted as treatment benefit. If post-treatment ctDNA decline, tumour shrinkage or immune-related adverse events are used as baseline predictors, predictive information is mixed with mediating processes that occur after treatment initiation.

TTE is therefore not synonymous with advanced statistics; it is a design-first safeguard. A scoping review by Zuo and colleagues showed rapid growth in TTE across oncology, cardiovascular and infectious disease research, but also found that many studies did not adequately specify time zero, emulate random assignment or define treatment strategies, indicating that claiming TTE is not the same as completing a target-trial design (60). The TARGET statement translated these principles into a 21-item reporting checklist that requires authors to report both the target-trial protocol and its mapping to observational data (61). A methodological review in JASN similarly argued that TTE should become a standard approach for observational studies of intervention effects because it forces investigators to define strategies, follow-up windows and estimands before analysis (62). These papers are useful design references, but they are also a warning: a study can cite TTE terminology while remaining vulnerable if eligibility, time-zero and strategy definitions are incomplete.

Leakage checks should be explicit before model training. Endpoint leakage occurs when variables derived from future progression, death, toxicity grading or response assessment enter the feature set. Data leakage occurs when image normalization, feature selection or cross-validation is performed before train-test separation, allowing information from the validation cohort to influence the model. Treatment-pathway leakage occurs when variables such as number of ICI cycles completed, maintenance therapy, later-line treatment, follow-up scan frequency or survival to biopsy proxy the outcome or the treating institution rather than baseline eligibility. Post-treatment variable misuse occurs when early response, ctDNA decline, irAEs, steroid exposure or dose interruption are treated as baseline covariates instead of mediators or time-varying states. A target-trial data dictionary should label every candidate feature as pre-treatment, at-index, post-treatment, outcome-derived or administrative before analysis.

### 5.3 Translating immunotherapy questions into target trial protocols

Applying TTE to immunotherapy requires specification of at least six elements. First, the target population must match a real decision setting, such as first-line advanced NSCLC, whether to add immunotherapy after SABR in early inoperable NSCLC, whether to extend perioperative immunotherapy or whether to stop treatment after durable response. Second, time zero must be the shared decision point at which all candidate strategies are possible, not a post-treatment event determined by outcome. Third, strategies should be operationalized, for example immediate pembrolizumab monotherapy, pembrolizumab plus platinum chemotherapy, treatment for up to 2 years or until unacceptable toxicity, or discontinuation after complete response with close monitoring. Fourth, follow-up and outcomes should be prespecified, including overall survival, progression-free survival, restricted mean survival time, pathological complete response, immune-related adverse events, treatment interruption, quality of life or composite net benefit. Fifth, the causal contrast should distinguish intention-to-treat, per-protocol and as-treated estimands. Sixth, the analysis plan should specify confounding adjustment, censoring, switching, missing data and sensitivity analyses. The TARGET statement operationalizes these requirements and improves transparency for peer review and clinical interpretation (61).

This protocol-based approach improves AI studies by assigning each feature a role before modelling. Radiomic, digital pathology, multi-omics and LLM-derived features can support confounding adjustment by capturing tumour burden, organ function, inflammatory state and prior therapy; identify candidate effect modifiers such as tumour-infiltrating lymphocytes, microbiome profiles or imaging heterogeneity; or support dynamic updating when a new target trial is defined at a landmark time, for example to compare continuation, switching, local therapy or discontinuation.

For long-follow-up immunotherapy studies, treatment switching, discontinuation, censoring and changing standards of care should be addressed in the estimand rather than handled *post hoc*. Intention-to-treat estimands estimate the effect of initiating a strategy despite subsequent switching; per-protocol estimands require explicit adherence definitions and clone-censor-weight or inverse-probability-of-censoring methods; as-treated estimands require time-varying exposure models and careful interpretation. Calendar time should be considered when PD-L1 thresholds, molecular testing, perioperative indications or combination regimens change during follow-up. Sequential emulation, calendar-stratified analyses and sensitivity analyses for informative censoring are essential when immunotherapy practice evolves.

Missing data require a causal interpretation rather than a purely technical imputation step. Biomarker, imaging, molecular and microbiome measurements are often missing because a test was not clinically indicated, tissue was insufficient, a patient was too unwell to undergo imaging, the assay was unavailable, or follow-up differed by response and toxicity. These mechanisms may be missing completely at random, missing at random conditional on observed clinical history, or missing not at random because unobserved disease severity or access to care drives measurement. Immunotherapy TTE studies should report modality-specific missingness, distinguish absent testing from negative results, use multiple imputation, inverse-probability-of-observation weights or joint modelling when appropriate, and perform sensitivity analyses for not-at-random mechanisms.

### 5.4 Early oncology and immunotherapy examples

Recent immunotherapy studies have begun to combine machine learning with target trial emulation. A JCO abstract in advanced urothelial cancer used Flatiron Health real-world electronic health record data to compare first-line pembrolizumab with gemcitabine and carboplatin in a population resembling KEYNOTE-361, and used machine-learning phenotyping to identify patients at high risk of early death who were less likely to benefit from the delayed effect of checkpoint inhibition. After exclusion of the highest-risk tertile, the pembrolizumab group showed clearer advantages in 3-year survival and 4-year restricted mean survival time (63). This is a useful methodological example because phenotyping was embedded in a treatment comparison, but it should be interpreted cautiously: abstract-level reporting limits assessment of variable validity, missingness, residual confounding and transportability to other EHR systems.

Target-trial thinking can also be combined with mechanistic models and virtual population inference. Pawlowski and colleagues used machine-learning surrogate models to accelerate virtual population inference in immuno-oncology quantitative systems pharmacology (QSP), allowing virtual trials to explore larger parameter spaces and fit tumour-size distributions from pembrolizumab trials (64). QSP-based simulation differs from real-world TTE: the former emphasizes mechanism and virtual populations, whereas the latter estimates causal effects from observational patient data. The two approaches are complementary. TTE anchors real-world comparisons in a clinically meaningful design and bias-control framework; QSP and digital twins can explore mechanistic plausibility, dose and timing, and virtual treatment trajectories.

### 5.5 What TTE adds to causal AI in immunotherapy

In practice, TTE separates design from estimation. The same estimator can answer different questions depending on time zero, treatment strategy, adherence and censoring rules; conversely, no estimator can rescue a cohort in which patients enter only after early progression, toxicity or treatment switching has already occurred. Section 5 therefore considers methods only after these design decisions have been made.

## 6 Treatment-effect learning methods for heterogeneous and dynamic immunotherapy decisions

**Figure 4:**
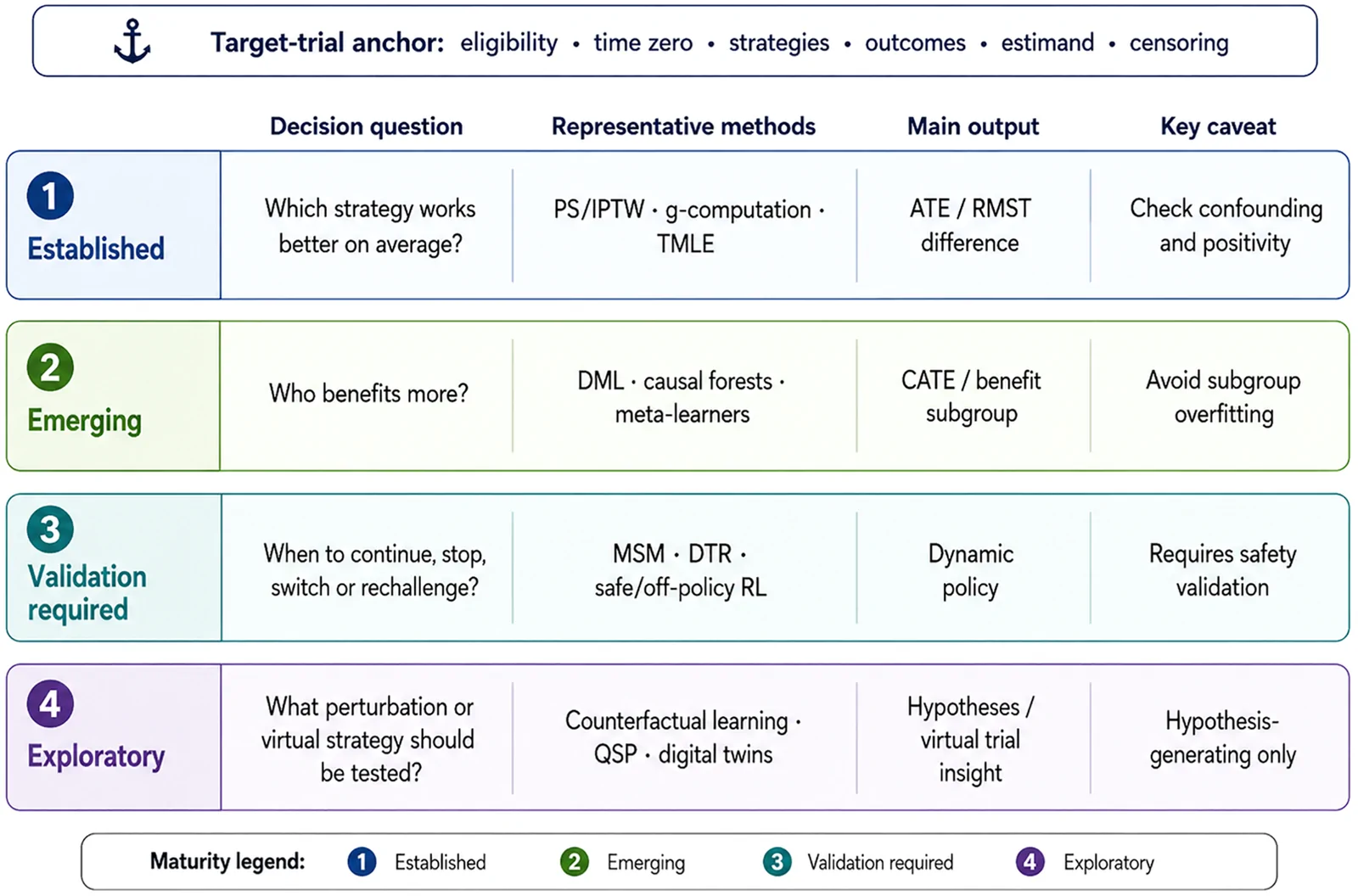
Matching immunotherapy decision questions to causal AI method maturity. Causal AI methods in immunotherapy should be selected according to the underlying decision question and anchored to a target trial specifying eligibility, time zero, treatment strategies, outcomes, estimand and censoring. Established baseline-comparison methods (PS/IPTW, g-computation and TMLE) estimate average treatment effects such as ATE or RMST differences. Emerging heterogeneous-treatment-effect methods (DML, causal forests and meta-learners) identify subgroups more likely to benefit. Validation-required dynamic-strategy methods (MSMs, DTRs and safe/off-policy RL) address sequential decisions such as continuation, switching, discontinuation and rechallenge. Exploratory methods (counterfactual learning, QSP and digital twins) mainly support hypothesis generation or virtual-trial insight and should not be treated as standalone causal evidence.

If Section 4 defines the trial to be emulated, Section 5 addresses how treatment effects can be estimated within that framework. Immunotherapy strategy optimization typically faces three challenges: treatment is not randomly assigned, benefit is heterogeneous across patients and decisions are updated over time. The role of causal AI is not to replace causal assumptions with machine learning. Rather, under a clear target trial and identification assumptions, machine learning can help model high-dimensional covariates, nonlinear effect modification, complex censoring and dynamic strategies. The method landscape for baseline, heterogeneous, dynamic and simulation-based questions is summarized in **Figure 4**.

### 6.1 Controlling measured confounding: propensity scores, g-methods, and TMLE

In non-randomized immunotherapy studies, the first challenge is measured confounding. Propensity-score methods can support comparisons of ICI monotherapy versus combination therapy or immunotherapy plus local therapy versus local therapy alone, provided key determinants of treatment and outcome are measured before time zero, including stage, tumour burden, ECOG performance status, organ function, laboratory values, molecular biomarkers, prior therapy and comorbidities (66).

When treatment, response and toxicity evolve over time, simple baseline adjustment may be biased. Marginal structural models, g-computation and targeted maximum likelihood estimation can address longitudinal strategies, time-varying confounding and doubly robust effect estimation under appropriate identification assumptions (67–69). These methods are directly relevant when early imaging response, ctDNA change, corticosteroid use, organ function or irAEs influence later treatment adaptation and long-term outcomes.

Machine learning can improve nuisance-function estimation for propensity, outcome or censoring models, but it cannot remove unmeasured confounding, repair an erroneous time zero, overcome structural positivity violations or fix poorly defined outcomes. Exchangeability means that, conditional on measured baseline history L0, treatment assignment is as good as random for the target estimand. High-dimensional AI phenotypes may strengthen exchangeability if they capture tumour burden, immune context, frailty or access pathways that otherwise confound treatment choice; they may also weaken credibility if they are opaque proxies for institution, scanner, sequencing platform or clinician preference. Positivity means that every clinically relevant covariate pattern has a non-zero probability of receiving each strategy. Radiomics or pathology models that create hundreds of spatial strata can expose practical positivity problems when some algorithm-defined strata almost always receive one regimen. TMLE can combine flexible outcome and treatment models with a targeting step for the prespecified estimand, but it still requires overlap diagnostics, truncation or collaborative covariate selection when propensity scores are extreme. DML uses sample splitting, cross-fitting and orthogonal scores to reduce overfitting bias in high-dimensional adjustment, but it also requires adequate overlap, prespecified nuisance learners and sensitivity analyses for unmeasured confounding. In this setting, AI assists causal estimation; it does not replace causal design.

### 6.2 Estimating heterogeneous treatment effects with causal machine learning

The most clinically relevant immunotherapy questions often concern heterogeneous treatment effects rather than average effects. Double or debiased machine learning (DML) uses sample splitting, cross-fitting and orthogonalization to combine high-dimensional machine learning with treatment-effect estimation while reducing overfitting bias (70). This is well suited to multimodal immunotherapy data, in which imaging, pathology, omics and EHR variables far exceed the dimensionality that conventional regression can handle reliably. DML can estimate overall effects and can also provide a foundation for causal forests, meta-learners and other conditional average treatment-effect models.

Causal forests and generalized random forests extend random forests to local treatment-effect estimation, allowing models to learn which regions of a high-dimensional covariate space have larger conditional average treatment effects (CATEs) (71, 72). Meta-learners, including S-, T-, X- and R-learners, provide a flexible framework for turning supervised learners such as gradient boosting, random forests, neural networks or survival models into treatment-effect estimators (73). For censored outcomes such as overall and progression-free survival, causal survival forests extend heterogeneous treatment-effect estimation to right-censored time-to-event data, making them particularly relevant for oncology (74).

These methods offer a route from biomarker stratification to effect stratification. Radiomics, tumour-infiltrating lymphocytes, MSI, ctDNA, microbiome and single-cell features should not only be used to predict who will progress. They can also serve as candidate effect modifiers for estimating which features increase the incremental benefit of combination therapy over monotherapy. Interpretation of CATE models requires caution. Subgroup effects are vulnerable to small samples, model selection, censoring mechanisms and multiple exploration. Without a target-trial protocol, external validation and decision-curve analysis, highly individualized effect estimates may reflect data-driven chance patterns rather than clinically actionable heterogeneity.

### 6.3 Dynamic treatment regimes and reinforcement learning

Many immunotherapy decisions are not made once at baseline; they are repeatedly updated during treatment. Murphy’s framework for optimal dynamic treatment regimes defines treatment strategy as a sequence of decision rules based on patient history, providing a statistical basis for deciding when to continue, stop, combine or switch treatment (75). In immunotherapy, dynamic strategies may be defined at several decision points: baseline choice of monotherapy or combination therapy; early imaging or ctDNA change and possible intensification; interruption or rechallenge after immune-related adverse events; discontinuation after durable response; and continuation of ICIs with local therapy after progression.

The structural shift from baseline g-computation to dynamic landmark or sequential target-trial modelling is essential. Standard g-computation for a baseline treatment estimates E[Y^a] using covariates measured before t0. Dynamic treatment regimes instead define decision times k = 0, 1,…, K, histories Hk = (L0, A0, L1, A1,…, Lk), and rules gk(Hk) that map the observed history to an action such as continue, interrupt, switch, add local therapy or rechallenge. At each landmark, eligibility, treatment options and outcomes are redefined using only information observed up to that landmark; future response, toxicity or survival information cannot enter the rule. Estimation then uses sequential g-computation, marginal structural models, Q-learning, A-learning or nested target-trial emulations with censoring weights so that conditional adaptations are evaluated without converting mediators into baseline covariates.

Reinforcement learning (RL) provides a computational language for dynamic treatment strategies, but in medicine it must be constrained by safety and causal principles. Guidelines for RL in healthcare published in Nature Medicine emphasize off-policy evaluation, confounding, unobserved states, reward definition, safety constraints and prospective validation, and warn against treating policies learned from retrospective data as deployable clinical recommendations (76). Recent oncology studies illustrate the potential of combining RL with mechanistic models. A multiscale mathematical model-informed RL framework simulated dynamic interactions among glioblastoma cells, macrophages and treatment to optimize combination schedules (77). In stage IIB/IIC melanoma, an RL simulation comparing adjuvant nivolumab and pembrolizumab incorporated relapse-free survival benefit and penalties for severe adverse events, showing that treatment priority changes as toxicity weights change (78).

These examples show that the value of RL is not to name a best drug, but to place efficacy, toxicity, time and patient state within one strategy-optimization problem. In immunotherapy, rewards should not be limited to tumour shrinkage or progression-free survival. They should also reflect immune-related adverse events, treatment interruption, quality of life, downstream treatment opportunities and cost. However, off-policy RL from retrospective EHRs is especially vulnerable to embedded physician-behaviour bias: the historical data encode access to molecular testing, local drug availability, surveillance frequency, clinician risk tolerance and institutional toxicity-management habits. If these loops are not modelled, an RL policy can reproduce treatment-access disparities or conservative institutional practice rather than biological treatment utility. RL policies must therefore be linked to target trials, action-set positivity, clinically meaningful rewards, counterfactual off-policy evaluation, safety constraints, expert review and prospective silent-mode testing. Without these safeguards, RL may learn infeasible or harmful strategies.

### 6.4 Counterfactual learning, spatial perturbation, and treatment design

Beyond estimating effects of existing strategies, causal AI can help generate new interventional hypotheses. A counterfactual learning study in Nature Biomedical Engineering used spatial proteomic profiles of tumours to identify minimal perturbations predicted to increase T-cell infiltration, revealing combinatorial molecular changes associated with T-cell entry across cancer types (79). Such methods complement counterfactual tissue generation discussed in Section 3. They do not directly prove that a clinical treatment will work, but they can identify potentially actionable spatial immune mechanisms for drug combinations, target selection and translational experiments.

The immunotherapy design space also includes antigen and cell-therapy optimization. Deep reinforcement learning for MHC class I peptide generation and tumour-immune microenvironment models for ICI-chemotherapy scheduling illustrate how AI can propose candidate interventions or schedules (80, 81). These outputs should be viewed as upstream hypotheses that require experimental validation and subsequent target-trial or prospective clinical comparison.

Digital twins extend this idea toward patient-specific virtual counterparts that simulate feasible strategies under uncertainty. Reviews in solid tumours and CAR-T therapy propose integration of imaging, omics, pathology, clinical time series and mechanistic models to explore treatment trajectories, toxicity and trial design (82, 83). In this Review, digital twins are explicitly treated as exploratory boundary simulations: they can stress-test assumptions, prioritize strategies and visualize uncertainty, but they are not parallel causal evidence structures and should not be presented as patient-specific proof that one clinical strategy is superior.

Counterfactual perturbation, RL-based candidate generation and digital twins cannot bypass causal validation. A model may propose perturbations that increase T-cell infiltration, generate peptides with higher predicted MHC binding or simulate a patient’s trajectories under different treatments (84). These outputs still require experimental validation, target-trial clinical comparison and prospective evaluation. Their most appropriate role is to expand the space of treatment hypotheses and individualized simulations, not to replace clinical evidence.

### 6.5 Matching methods to immunotherapy decision questions

Method choice should be driven by the clinical question, not by the novelty of the model or the data modality. If the question concerns the average effect of two baseline strategies, propensity-score methods, inverse-probability weighting, g-computation or targeted maximum likelihood estimation may be sufficient. If the question is which patients are better candidates for combination therapy, DML, causal forests, meta-learners or causal survival forests are more appropriate. If the question involves on-treatment response, toxicity and decisions after discontinuation, marginal structural models, dynamic treatment regimes or reinforcement learning may be needed. If the goal is to discover immune-modulating mechanisms or build virtual trials, counterfactual learning, QSP surrogates and digital twins may be most useful. **Supplementary Table 1** extends this method-to-question mapping and lists representative algorithms, assumptions, outputs and caveats for each method family.

## 7 Clinical use cases in immunotherapy strategy optimization

Section 6 applies the framework to six recurring immuno-oncology decisions: treatment intensification, perioperative de-escalation or organ preservation, radiotherapy-immunotherapy balance, irAE management, discontinuation or rechallenge, and weak-evidence populations. For each setting, the key issue is whether cited AI studies define a clinically executable action and evaluate evidence strong enough to support that action.

### 7.1 Combination immunotherapy and patient enrichment

Combination therapy is the clearest setting for immunotherapy strategy optimization. ICIs combined with chemotherapy, anti-angiogenic therapy, radiotherapy, targeted therapy or new immune agents can increase response rates, but also add toxicity, cost and treatment burden. Biomarker analyses from the phase III CONTINUUM and DIPPER trials in nasopharyngeal carcinoma provide a representative example. Investigators used RNA sequencing and metabolism-related genes to define molecular subtypes and applied a machine-learning classifier in validation cohorts to identify metabolic subtypes more likely to gain event-free survival benefit from PD-1 inhibition plus standard chemoradiotherapy (85). The randomized treatment context makes this evidence stronger than a single-arm response model, but its generalizability is still bounded by tumour type, regimen, biomarker platform and endpoint. Before similar molecular classifiers guide intensification in other cancers, they require calibration, prespecified subgroup testing, clinical-utility analysis and prospective confirmation that the added toxicity and cost are justified by incremental benefit.

### 7.2 Perioperative and organ-preserving immunotherapy

Perioperative immunotherapy is another setting in urgent need of strategy optimization. Neoadjuvant immunotherapy or chemoimmunotherapy can increase major pathological response (MPR) and pathological complete response (pCR), but sensitivity varies widely, and overtreatment may delay surgery, increase perioperative risk or cause irreversible toxicity. NeoPred used dual-phase CT and deep learning to predict MPR and pCR after neoadjuvant chemoimmunotherapy in resectable NSCLC, turning routine imaging from a static staging tool into a proxy for preoperative pathological response (86). In oesophageal squamous-cell carcinoma, medical imaging AI has also been used to support precision neoadjuvant immunochemotherapy by identifying patients likely to achieve deep pathological response, thereby informing intensification, surgical timing and potential organ preservation (87).

Perioperative AI must nevertheless avoid interpreting pathological-response prediction as proof of an optimal treatment strategy. MPR and pCR are important intermediate endpoints, but whether they substitute for long-term event-free survival, overall survival or quality of life depends on tumour type, regimen and follow-up. More importantly, if preoperative imaging or pathology models are to guide delayed surgery, treatment extension or organ preservation, the target trial must be explicit. For example, among patients who achieve deep radiological response after two cycles of neoadjuvant chemoimmunotherapy, investigators might compare planned surgery, additional cycles before surgery and close observation or organ preservation. Only within such a framework can AI-predicted pathological response become an evaluable clinical action.

The same logic suggests that early drug development should move from population-average signals toward mechanism-driven enrichment. The Society for Immunotherapy of Cancer has argued that failures of new immuno-oncology agents in early or late trials do not necessarily mean that a target is ineffective; they may reflect heterogeneity in tumour immune state, treatment history, antigen load, spatial immune architecture and concomitant therapy (88). Enrichment designs should therefore integrate mechanism, measurable biomarkers, pharmacodynamic readouts and target-trial comparisons early in development, so that potential benefit in responsive subgroups is not diluted by average effects. For causal AI, the priority is to support trial enrichment, subgroup-effect exploration and prospective stratification, not *post hoc* discovery of complex patterns that are difficult to validate.

### 7.3 Toxicity-aware treatment selection and irAE phenotyping

Immunotherapy strategy optimization must estimate toxicity, treatment discontinuation and long-term quality of life alongside efficacy. Immune-related adverse events (irAEs) can affect multiple organs, emerge at different time points and vary widely in severity. They may indicate immune activation, but can also lead to treatment interruption, corticosteroid use, hospitalization or death. A multicancer ICI cohort study of germline predictors of treatment discontinuation due to irAEs suggests that host genetics may help identify patients more likely to stop treatment because of severe toxicity (89). A scoping review of AI for ICI safety found studies using EHRs, imaging, laboratory values, clinical text and multi-omics data, but most remained retrospective risk-prediction or event-detection studies with limited prospective validation, interpretability and workflow integration (90).

Toxicity ascertainment itself is a key target for AI. Sun and colleagues compared LLMs with International Classification of Diseases codes for identifying severe irAEs in clinical records and found that natural-language models could capture toxicities that structured codes often miss, improving the data basis for real-world safety research (65). In lung cancer, systematic reviews and meta-analyses of machine-learning models for radiation pneumonitis and checkpoint-inhibitor pneumonitis suggest that radiomics, dosiomics and clinical variables may improve risk identification, although included studies often have high risk of bias and limited external validation (91). Federated radiomics models have also been used to adapt pneumonitis prediction across centres without sharing raw data, supporting multicentre safety modelling for combined radiotherapy and immunotherapy (92). Gut microbiota and metabolite signatures have been associated with severe irAEs in advanced hepatobiliary cancers, suggesting that host ecology may influence both efficacy and toxicity (93).

These studies indicate that safety AI should not function only as a toxicity alarm. It should feed into net-benefit modelling. For the same patient, high probability of efficacy and high toxicity risk may coexist. Strategy selection requires comparison of regimens across efficacy, severe irAEs, discontinuation, downstream treatment opportunities and patient preferences. Dynamic treatment regimes and reinforcement-learning frameworks can contribute here by updating strategy during treatment on the basis of imaging, ctDNA, laboratory measures, early irAE signals and patient-reported outcomes, and by comparing the expected net benefit of continuation, interruption, dose modification, immunosuppression, switching or rechallenge.

### 7.4 Radiotherapy-immunotherapy combinations and pneumonitis-aware optimization

Radiotherapy-immunotherapy combinations exemplify the interaction between local treatment and systemic immune modulation. Radiotherapy can, in principle, enhance immune response through antigen release, inflammatory signalling and remodelling of the tumour microenvironment. Clinically, however, thoracic radiotherapy combined with ICIs can increase pneumonitis risk and narrow the therapeutic window. Habitat radiomics analyses in NSCLC have shown that tumour and peritumoural imaging habitats can be associated with both progression-free survival and immune-related adverse reactions, suggesting that spatial imaging heterogeneity may support joint efficacy-toxicity modelling (94). Consistent with the argument in Section 3, the value of such models is not to predict progression-free survival or pneumonitis in isolation, but to support net-benefit comparisons among ICI alone, concurrent or sequential radiotherapy plus ICI, SABR after oligoprogression and avoidance of thoracic combination therapy in high-risk patients.

AI studies of radiotherapy-immunotherapy should therefore adopt a target-trial framework. Time zero should be the clinical decision point for combining radiotherapy and immunotherapy. Dose, irradiated volume, lung dosimetry, ICI agent and treatment interval should be operationalized. Outcomes should include local control, progression-free and overall survival, grade 3 or higher pneumonitis, treatment discontinuation and quality of life. Without these definitions, models may misclassify patients who survive long enough to complete combination therapy and undergo reassessment as treatment beneficiaries, introducing immortal time and selection bias.

### 7.5 Treatment duration, discontinuation, and rechallenge

As more patients achieve durable responses, treatment duration, discontinuation and rechallenge have become central questions in the later course of immunotherapy. In current practice, ICIs are often stopped after 2 years, unacceptable toxicity, disease progression or patient preference, but there is no stable algorithm for identifying who can stop safely. A multimodal study by Noh and colleagues showed that clinical, imaging, pathological and molecular features can predict relapse after ICI cessation in advanced melanoma, illustrating the need to integrate multiple data layers in discontinuation decisions (95). From a causal perspective, however, prediction of relapse after stopping is not the same as estimating whether continued treatment would reduce relapse. The latter requires a new target trial among patients who have reached a defined response state, comparing continuation, planned discontinuation and discontinuation with monitoring or rechallenge.

A useful contrast is relapse prediction after ICI discontinuation versus a target trial of continuation. A flawed framing trains a model only among patients who already stopped treatment and asks who relapsed; such a model may be useful for surveillance but cannot estimate whether continuing ICIs would have prevented relapse. An improved framing defines time zero among durable responders at a prespecified landmark, such as 24 months of therapy or confirmed complete/partial response, then compares executable strategies: continue ICI, planned discontinuation with monitoring, or discontinuation with prespecified rechallenge. The estimand might be the intention-to-treat difference in 24-month relapse-free survival, RMST, recurrent irAEs or toxicity-weighted utility. Post-discontinuation ctDNA, scans and symptoms are then outcomes or landmark state variables, not baseline covariates.

Drug interactions and concomitant medications further complicate discontinuation and rechallenge decisions. Reviews of immunological drug-drug interactions indicate that antibiotics, corticosteroids, proton-pump inhibitors, immunosuppressants and targeted agents may affect ICI efficacy and toxicity through the microbiome, immune-cell state or pharmacokinetics, and AI may help identify complex interaction patterns in real-world data (96). These interactions also require causal design. Many concomitant medications are markers of disease severity, infection, irAEs or comorbidities. Without addressing confounding by indication, models may mistake clinical status for drug effects.

### 7.6 Real-world weak-evidence populations and pragmatic risk-benefit tools

Many real-world patients do not meet the eligibility criteria of pivotal randomized trials, including older adults and patients with autoimmune disease, organ dysfunction, brain metastases, infection risk, malnutrition or rare tumour types. Risk-prediction models for patients with HCC receiving atezolizumab-bevacizumab and nutrition-inflammation nomograms for recurrent or metastatic cervical cancer treated with ICIs illustrate the need for clinical-variable modelling in weak-evidence populations (97, 98). These tools may be useful for triage and risk communication, but their decision value is limited unless they distinguish baseline frailty, access to care and tumour biology from modifiable treatment benefit. In these populations, transportability, competing risks, patient preference, supportive-care optimization and equity should be assessed before risk scores are used to intensify or withhold immunotherapy. A full clinical use-case matrix mapping immunotherapy decisions to candidate strategies, causal frameworks, endpoints, validation needs and common misuses is provided in **Supplementary Table 2**.

## 8 Validation, implementation, and regulatory considerations

The clinical scenarios above show where causal AI and target trial emulation may be useful, but the path from research model to clinical tool remains long. npj Digital Medicine is concerned not only with novelty, but with whether a model is reproducible, interpretable, validated, deployable and able to improve clinical outcomes. Translation is particularly difficult in immunotherapy because data modalities are complex, treatment standards change rapidly, outcomes may be delayed, toxicity can affect multiple organs and model outputs may influence high-cost, high-risk decisions. Validation, implementation and regulation should therefore be built into the causal question and target-trial workflow from the start.

### 8.1 Transparent reporting and risk-of-bias assessment

Transparent reporting is the basis for evaluating clinical AI. TRIPOD+AI updates traditional TRIPOD guidance for diagnostic and prognostic models by adding information relevant to machine learning, including data sources, data splits, feature processing, tuning, code availability, human-AI interaction and reproducibility (99). PROBAST+AI extends risk-of-bias and applicability assessment for prediction models to AI settings, emphasizing participant selection, predictor definition, outcome measurement, sample size, missing data, model development and validation (100). These standards are particularly important for immunotherapy AI, where small samples, high-dimensional features, retrospective labels, single-centre training, limited external validation and inconsistent endpoints are common.

TRIPOD+AI and PROBAST+AI mainly address prediction models. If a model claims to support treatment selection or treatment-effect estimation, it should also report the target-trial protocol, time zero, treatment strategies, causal estimand, confounding control, positivity, censoring procedures and sensitivity analyses. Prediction-model reporting answers whether the model has been described adequately. Target-trial and causal-inference reporting answer whether the question has been defined correctly. Both are minimum requirements for transparent causal AI in immunotherapy.

### 8.2 Prospective evaluation and AI-specific clinical trial reporting

Retrospective performance is not a substitute for clinical evaluation. CONSORT-AI and SPIRIT-AI extend clinical trial reports and protocols for AI interventions by requiring clear description of the AI system’s inputs, outputs, setting of use, version, error handling, human operator role and influence on clinical decisions (101, 102). DECIDE-AI focuses on early clinical evaluation and recommends documenting usability, workflow fit, clinician trust, failure modes and unintended consequences before a full randomized trial of AI decision support (103). In immunotherapy, these standards mean that an AI tool should not report only offline AUC or CATE strata. It should specify when it appears in a multidisciplinary tumour board or clinic workflow, how it changes recommendations, whether clinicians can override it and how erroneous recommendations will be monitored.

Work on clinical AI translation repeatedly shows that models often fail to produce clinical impact not because the algorithm is too simple, but because training data do not match the target population, the model is not embedded in workflow, action thresholds are unclear, accountability is undefined or prospective outcome evaluation is absent (104). In immunotherapy, the consequences of model-guided action are direct: underestimating benefit may deprive patients of long-term survival opportunities, whereas overestimating benefit may expose them to ineffective treatment and severe irAEs. Prospective evaluation of causal AI tools should therefore consider pragmatic trials, stepped-wedge designs, cluster randomization or prospective silent trials that first observe the discrepancy between model recommendations and clinical decisions before testing the effect of acting on the model.

### 8.3 Calibration, decision curves, and clinical utility

Validation should not stop at discrimination. Van Calster and colleagues described calibration as the Achilles heel of predictive analytics because even models with high AUC can systematically overestimate or underestimate event probabilities in clinically important risk ranges (105). Immunotherapy decisions often occur near thresholds, such as whether to add chemotherapy for marginal benefit, avoid thoracic radiotherapy because of high pneumonitis risk or stop therapy after durable response. Poor calibration in these regions can lead to wrong action despite good overall performance.

Decision-curve analysis provides a way to evaluate clinical net benefit by weighting true-positive benefit and false-positive harm across clinical thresholds, and is useful for comparing strategies such as use the model, treat all or treat none (106). For causal AI, decision-curve analysis can be extended to strategy-level utility: it can assess not only whether a model predicts irAEs or relapse, but whether model-guided intensification, discontinuation or rechallenge improves patient-level utility. Future immunotherapy models should report discrimination, calibration, clinical net benefit, subgroup stability, fairness and impact on treatment recommendations rather than a single performance metric.

For treatment-effect models, clinical utility also requires predefined action thresholds and a transparent utility function. Investigators should specify how survival gain, grade 3 or higher irAEs, quality of life, treatment burden, downstream treatment options and patient preferences are weighted; whether the tool is intended for clinician-facing decision support rather than autonomous recommendation; and how multidisciplinary teams can override recommendations. Silent-mode or prospective workflow studies should document whether model output changes decisions and whether those changes improve net benefit.

### 8.4 External validation, harmonization, and transportability

Cross-centre generalization is a central challenge for immunotherapy AI. AI-based MSI screening studies suggest that digital pathology can be a cost-effective entry point for immunotherapy-related biomarker assessment, but clinical value depends on reliability across cancer types, staining workflows and scanners (107). A two-centre CT-based interpretable deep-learning signature for PD-L1 expression in bladder cancer suggests that interpretability may improve clinical acceptability, but broader multicentre prospective validation is still needed (108). Longitudinal radiomics studies show that CT harmonization can substantially affect immunotherapy response prediction in NSCLC, indicating that preprocessing, reconstruction parameters and centre effects can change model outputs (109).

These issues are even more consequential for causal AI. A CATE model or dynamic policy that appears useful in one centre may not transport to another because treatment access, imaging frequency, follow-up patterns, PD-L1 testing platforms, sequencing workflows and toxicity management differ. External validation should therefore examine not only prediction performance, but also covariate distributions, positivity, treatment-strategy definitions, stability of effect estimates and clinical transportability. Recalibration, domain adaptation, federated learning or target-population weighting may be required rather than assuming direct transfer across centres.

When follow-up spans multiple immunotherapy eras, analyses should document calendar-time changes in standards of care, assays and access. Eligibility criteria, exposure definitions, PD-L1 or MSI testing, sequencing workflows, local therapy, corticosteroid practice and later-line options may change after model development. Calendar-stratified TTE, rolling external validation, transportability weighting and prespecified re-estimation can help distinguish biological treatment heterogeneity from secular practice change.

### 8.5 LLMs, evidence extraction, and decision support governance

LLM and retrieval-augmented generation (RAG) tools are entering oncology evidence extraction and decision support. SEETrials uses LLMs to extract safety and efficacy information from oncology clinical trials, illustrating how automated extraction may help researchers and clinical teams update trial evidence more rapidly (110). Time-series clinical models such as CAAN-LSTM use context-aware adaptive normalization to handle temporal dependence in immunotherapy clinical data and provide structured decision support (111). These tools can support TTE by extracting real-world variables, treatment lines, outcomes and toxicities, or by drafting target-trial protocols for expert review.

LLM-based decision support requires governance. Treatment recommendations should be traceable to guidelines, trials, real-world evidence or local validation. LLM outputs should not bypass multidisciplinary teams, oncology specialists or shared decision-making with patients. Systems need to record versions, prompts, retrieval sources, uncertainty and human overrides. If LLMs are used to draft target trials, screen variables or explain causal models, their role should be defined as literature and data-management support, not as causal effect estimation.

### 8.6 Regulatory framing and lifecycle monitoring

Regulation of immunotherapy AI is challenging because model outputs may affect drug choice, treatment duration, discontinuation, rechallenge and toxicity management. If a model is used as clinical decision-support software, its intended use, target population, input data, output interpretation, contraindicated scenarios and human oversight should be clear. Continuously learning models also require version control, drift monitoring, performance revalidation and update governance. Rapid changes in treatment standards can produce evidence drift: a model trained in an older first-line era may not apply to neoadjuvant or combination-therapy settings.

Clinical implementation should therefore include lifecycle monitoring from development through deployment. Before deployment, models should undergo external validation, bias assessment, calibration and clinical utility analysis. During early implementation, silent or shadow mode can record model recommendations without affecting care. Once a model influences decisions, systems should monitor outputs, clinician adoption, patient outcomes, toxicity, subgroup fairness and possible harms. Causal AI can become a trustworthy tool for immunotherapy strategy optimization only if it improves net benefit in real workflows rather than merely improving offline metrics.

Clinical translation of causal AI requires prediction-model reporting standards, target-trial reporting, causal-inference transparency, external validation, clinical utility assessment and regulatory governance. The future directions most relevant to this Review are therefore those that strengthen evidence-to-protocol translation, bounded virtual simulation and privacy-preserving lifecycle learning rather than speculative model deployment.

## 9 Future perspectives: toward causal learning systems in immuno-oncology

Future immunotherapy AI should evolve from isolated predictors into governed learning systems that integrate real-world practice, trial evidence, mechanistic models and patient preferences under explicit target-trial and regulatory constraints. Three directions are most relevant: evidence-to-protocol translation with RAG and LLMs, bounded virtual simulation with spatial AI, QSP and digital twins, and federated lifecycle learning across institutions.

### 9.1 RAG and agentic LLMs for evidence-to-protocol translation

RAG and LLMs can provide infrastructure for evidence management and target-trial protocol development in immunotherapy research. Tools such as RefAI show that GPT-powered retrieval-augmented generation can combine language-model synthesis with traceable biomedical literature retrieval for recommendation and summarization (112). For causal AI in immunotherapy, the value of RAG extends beyond review writing. It could help translate evolving trials, guidelines, drug labels, real-world studies and safety alerts into structured research questions specifying the target population, treatment strategies, comparator, time zero, outcomes, follow-up and confounders.

Agentic LLMs may eventually serve as target-trial design assistants. They could identify whether a clinical decision problem is better suited to a randomized trial, real-world TTE, single-arm external-control analysis or mechanistic simulation; check alignment of time zero; flag possible immortal time bias, confounding by indication or misuse of post-treatment variables; and draft protocols, variable dictionaries and reporting checklists for expert review. Such systems must remain anchored in human expertise and traceable evidence. LLMs can accelerate the move from evidence to protocol, but they cannot replace causal assumptions, clinical feasibility assessment or ethical responsibility.

### 9.2 Spatial AI and foundation models for immune-context modelling

Spatial AI will be important for linking mechanism discovery with strategy optimization. Spatial transcriptomics, multiplex immunofluorescence, spatial proteomics and digital pathology can reveal how immune cells enter tumours, acquire excluded or exhausted states, and interact with tumour cells and stroma. Reviews of spatial AI in cancer argue that multimodal omics and deep learning can map the topology of immune evasion, moving the field from single biomarkers toward tissue-ecosystem representations of immune escape (113). This aligns with counterfactual spatial perturbation in Section 5: future models should not only identify patients likely to respond, but also explain which spatial structures might be intervened on to increase T-cell infiltration or reverse immune exclusion.

Foundation models will also reshape pathology and multimodal immune phenotyping. Vision-language pathology foundation models, clinical transformers and multi-omics foundation models may transfer across cancer types, tasks and small-sample settings, reducing the need to train each immunotherapy model from scratch. Federated learning for pan-cancer tumour-infiltrating lymphocyte detection suggests that multicentre collaboration can train more generalizable immune-infiltration detectors while preserving data privacy (114). These models still need to be made causal. Pretrained representations can enter TTE and CATE models as covariates or effect modifiers, but cross-domain representational similarity should not be interpreted as treatment benefit.

### 9.3 Digital twins and virtual trials for adaptive immunotherapy

Digital twins are among the most imaginative and most easily overpromised directions for causal AI. An immunotherapy digital twin would ideally integrate clinical time series, imaging, pathology, genomics, transcriptomics, microbiome, treatment exposures, toxicity and patient-reported outcomes, bridging mechanistic and data-driven models. The goal is not to create a perfect patient copy, but to simulate feasible alternative strategies under uncertainty: when to start combination therapy, when to reduce intensity or stop, whether to continue ICIs after progression, whether to rechallenge after irAEs and how to balance long-term survival with toxicity.

Digital twins and virtual trials can be combined with QSP surrogate models, dynamic treatment regimes and carefully constrained reinforcement learning to prioritize strategies for real-world TTE or prospective testing. They are also susceptible to hype and to hidden assumptions about tumour growth, immune activation, toxicity, adherence and access. Any virtual strategy should undergo external validation, calibration to real patient trajectories, sensitivity analysis, clinical expert review and testing against experimental or clinical data. A feasible path is to build partial, bounded digital twins for questions such as CAR-T expansion and cytokine-release syndrome risk, the number of perioperative immunotherapy cycles or the timing of discontinuation after durable response, rather than claiming full-course patient-specific causal evidence.

### 9.4 Emerging intervention design as hypothesis generation

AI-enabled neoantigen prediction, immunogenic peptide design and vaccine optimization illustrate how learning systems may generate candidate interventions rather than only compare existing regimens. Recent pipelines use LLM-enhanced or interpretable deep learning for personalized neoantigen prediction and expose shortcut bias in neoantigen models (115, 116). Complementary work applies systems biology, generative AI and mechanism-informed machine learning to immunogenic peptide, oncolytic peptide, epitope and peptide-MHCI binding prediction (117–120). Vaccine-oriented reviews and AI-assisted vaccine-development studies further frame neoantigen, circRNA and shared-antigen constructs as candidates for subsequent testing (121–123). However, offline antigen ranking or binding prediction does not establish patient benefit. Their role in this Review is therefore upstream hypothesis generation for future target trials, not treatment-effect estimation.

### 9.5 Federated, privacy-preserving, and continuously learning causal systems

The future of immunotherapy AI will also depend on balancing privacy protection with multicentre generalization. Single-centre models rarely cover the full diversity of populations, assay platforms, treatment pathways and follow-up systems. Direct pooling of raw data is often constrained by ethics, law and data governance. Federated learning, secure multiparty computation and distributed validation provide ways to train and evaluate models across centres. The federated radiomics and pan-cancer TIL examples discussed above show that privacy-preserving collaboration is feasible for imaging and pathology tasks, but this approach must extend to causal estimands, CATE models and dynamic strategy validation.

Continuously learning systems must also address model drift. Immunotherapy indications, combinations, adjuvant strategies and assays change rapidly; the treatment standard at the time of model training may be outdated by deployment. Causal learning systems should periodically re-evaluate target-trial questions, update variable definitions, monitor external validation performance, detect subgroup failure and pause or recertify models when necessary. They should also record whether model recommendations change clinician behaviour and whether those changes improve outcomes. Without this feedback loop, AI remains an offline research activity rather than part of a learning health system.

### 9.6 Roadmap from predictive models to causal digital medicine

A more mature evidence standard for immunotherapy AI should require four linked forms of appraisal. First, protocol validity: eligibility, time zero, strategies and estimands are clinically executable. Second, estimator validity: confounding, positivity, censoring, missingness and treatment switching are explicitly handled. Third, clinical utility: calibration, decision curves, toxicity-weighted net benefit and workflow impact are reported, not only discrimination. Fourth, lifecycle safety: transportability, model drift, fairness, recalibration and recertification are monitored as immunotherapy standards change.

**Figure 5:**
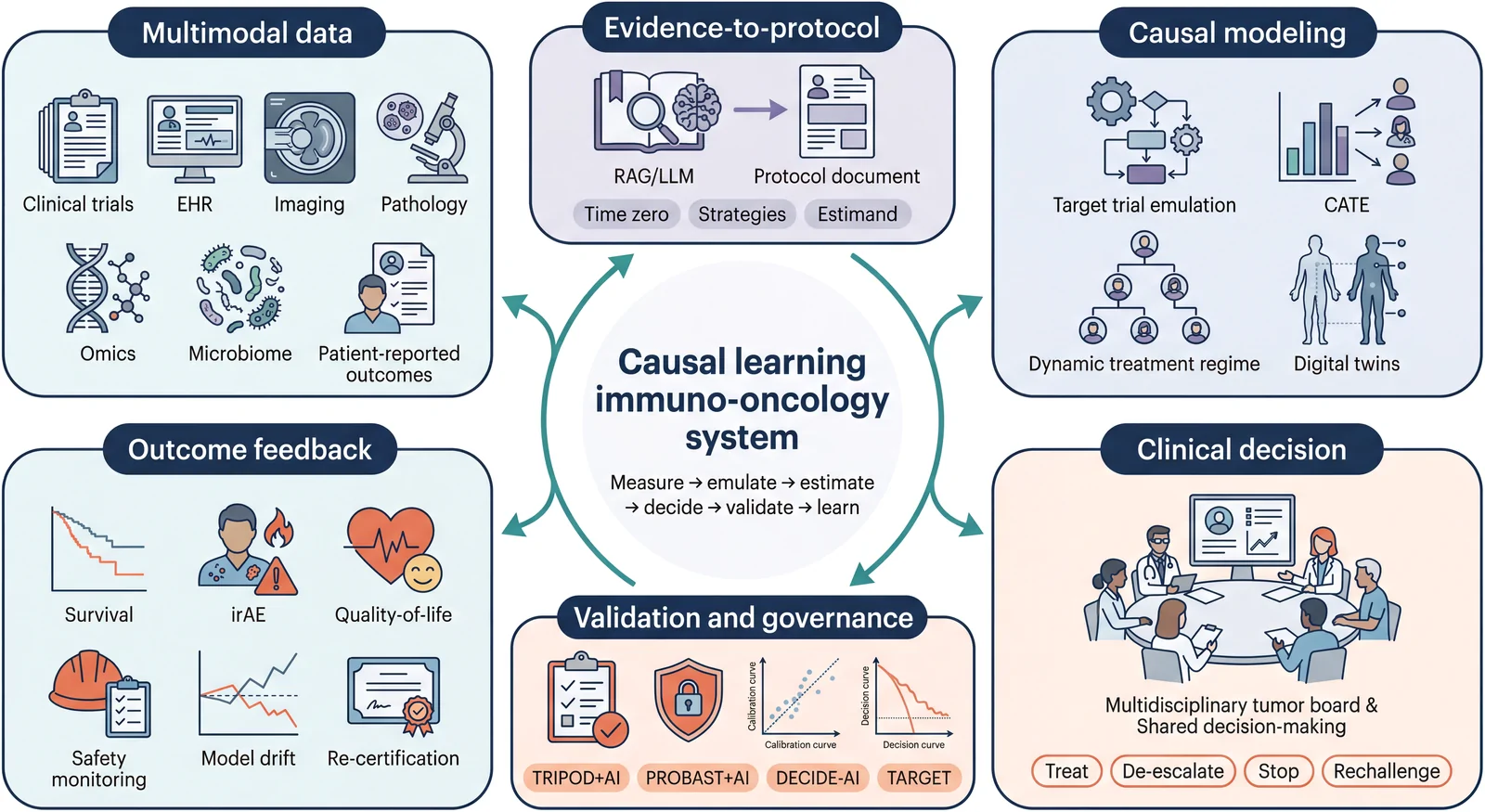
Implementation lifecycle for causal digital medicine in immunotherapy. Multimodal clinical-trial, EHR, imaging, pathology, omics, microbiome and patient-reported outcome data feed evidence-to-protocol translation, in which retrieval-augmented generation (RAG) and LLM workflows can help define time zero, strategies and estimands. Target trial emulation, CATE modelling, dynamic regimes and bounded digital-twin simulations support causal modelling. Established causal estimators should be separated from exploratory simulation and off-policy RL outputs. Before influencing care, outputs require calibration, decision-curve analysis, target-trial reporting, privacy governance, workflow evaluation and predeployment silent testing. After deployment, monitoring of clinician adoption, survival, safety, quality of life, fairness, drift, recalibration and re-certification closes the learning loop.

Thus, the endpoint of this field is a governed causal digital-medicine workflow rather than a single high-performing predictor. Such a workflow should compare feasible strategies, quantify uncertainty, balance efficacy and toxicity, and learn continuously within real clinical constraints. For example, in perioperative HCC, this decision-oriented logic could connect immunotherapy selection with AI-assisted surgical planning (124) and the choice between laparoscopic and robotic resection (125), treating these elements as components of an explicit multidisciplinary strategy rather than as isolated technical innovations. The proposed implementation lifecycle and learning loop are shown in **Figure 5**.
