# Supplementary material for: Causal AI for cancer immunotherapy: a narrative framework review of target trial emulation, treatment-effect learning and clinical translation
Source: Front Immunol. 2026 Aug 20;17:1896755. doi: 10.3389/fimmu.2026.1896755 (PMC13539561; doi:10.3389/fimmu.2026.1896755)
Supplement: Supplementary file 1 [file Table1.docx]

**Supplementary Materials**

**Supplementary Table S1 | Causal AI methods for immunotherapy strategy optimization**

| **Method family** | **Representative methods** | **Best-suited immunotherapy question** | **Required data / key assumptions** | **Main output** | **Major risks or caveats** |
| --- | --- | --- | --- | --- | --- |
| Propensity score methods | Matching, stratification, IPTW and overlap weighting [66] | Average effect of two baseline strategies, such as ICI monotherapy versus ICI plus chemotherapy, anti-VEGF therapy or radiotherapy | Sufficiently measured confounders; positivity; baseline covariates defined before time zero | ATE, ATT, risk difference, HR and RMST difference | Cannot address unmeasured confounding; extreme weights; biased if time zero is wrong |
| G-methods and longitudinal causal models | G-computation, marginal structural models and longitudinal IPTW [67-68] | On-treatment response, irAEs, ctDNA and steroids act as time-varying factors influencing future treatment and outcomes | Longitudinal covariate and treatment history; no unmeasured time-varying confounding; correct censoring handling | Regime-specific outcomes, standardized risks and effects of dynamic strategies | Model misspecification and temporal-order errors; post-treatment mediators and confounders must be separated |
| Doubly robust methods | TMLE, AIPW and super learner-assisted nuisance estimation [69] | Robust estimation of average treatment effects or standardized risks in real-world data | At least one of the treatment or outcome models is well specified; sample size supports flexible modelling | Doubly robust ATE, risk difference, RMST difference and confidence intervals | Fails if both nuisance models are poor or positivity is weak; more complex implementation |
| Double/debiased machine learning | DML, orthogonal learning and cross-fitting [70] | Confounding control or effect estimation with high-dimensional imaging, pathology, omics and EHR covariates | Sample splitting, cross-fitting, high-quality covariates and a clear estimand | Debiased treatment-effect estimates after flexible adjustment | Limited interpretability; cannot fix unmeasured confounding; requires adequate sample size and tuning |
| Heterogeneous treatment effect learning | Causal forests, generalized random forests and S/T/X/R-learners [71-73] | Which patients benefit more from treatment intensification, combination therapy or discontinuation? | Clearly defined treatment comparison; enough events; external validation; adequately measured candidate effect modifiers | CATE, benefit subgroups and ranked effect modifiers | Subgroup overfitting, multiple exploration and limited clinical interpretability; prognostic scores may be mistaken for benefit scores |
| Causal machine learning for survival outcomes | Causal survival forests and RMST-based causal models [74] | Heterogeneous treatment effects for right-censored OS and PFS outcomes | Modelable censoring process; consistent follow-up; clear endpoint definition | Time-to-event CATE, RMST differences and differences in risk curves | Informative censoring, competing risks and treatment switching complicate interpretation |
| Dynamic treatment regimes | Q-learning, A-learning, landmark TTE and dynamic treatment regimes [75] | When should therapy be continued, stopped, switched, rechallenged or combined with local treatment? | Sequential decision points; treatment history, state transition and outcome data; executable strategies | Individualized sequential treatment rules and dynamic net benefit | Complex treatment histories; strategies may be clinically infeasible; prospective evaluation is needed |
| Reinforcement learning | Off-policy RL, safe RL and model-informed RL [76-78] | Combination scheduling, toxicity-weighted treatment selection and adaptive treatment timing | Reliable reward definition, off-policy evaluation, safety constraints and rich longitudinal data | Policy, recommended action and simulated long-term reward | May learn physician-behavior and access-to-care bias from historical EHR loops; retrospective policies are not deployable without off-policy evaluation, safety constraints, expert review and prospective validation. |
| Counterfactual learning, QSP and digital twins | Spatial counterfactual learning, QSP surrogates, virtual populations and digital twins [79-84] | Discovery of actionable immune mechanisms, candidate peptides/combinations and virtual comparison of patient-specific treatment trajectories | Mechanistic priors, experimental validation, external calibration and uncertainty quantification | Candidate perturbations, virtual trial results and patient-specific simulations | Boundary simulation and hypothesis generation only; not parallel causal evidence. Experimental validation, target-trial emulation or prospective trials remain necessary. |

The method families are organized by the clinical decision they are best suited to address rather than by algorithmic novelty. All methods require a defined target trial, a prespecified estimand, adequate measurement of confounders, positivity, appropriate handling of censoring and external validation. Simulation-based tools and reinforcement learning should be considered decision-support or hypothesis-generating approaches until prospectively evaluated. Abbreviations: AIPW, augmented inverse probability weighting; ATE, average treatment effect; ATT, average treatment effect in the treated; CATE, conditional average treatment effect; DML, double/debiased machine learning; DTR, dynamic treatment regime; HR, hazard ratio; IPTW, inverse probability of treatment weighting; PS, propensity score; QSP, quantitative systems pharmacology; RL, reinforcement learning; RMST, restricted mean survival time; TMLE, targeted maximum likelihood estimation; TTE, target trial emulation.

**Supplementary Table S2 | Clinical use-case matrix for causal AI and target trial emulation in immunotherapy strategy optimization**

| **Clinical use case / core decision** | **Clinical use case / core decision** | **Candidate strategies** | **Causal framework** | **Primary endpoints** | **Key validation need** | **Common misuse to avoid** |
| --- | --- | --- | --- | --- | --- | --- |
| Combination therapy | Who needs intensification? | ICI alone vs chemoimmunotherapy or other combinations | RCT effect modification; TTE plus CATE | OS, PFS/EFS, RMST, grade >=3 irAEs, net benefit | External subgroup validation | Treating prognostic subtype as benefit subtype |
| Perioperative/organ preservation | Extend therapy, proceed to surgery or de-escalate? | Standard surgery pathway vs additional cycles or organ preservation | Landmark/sequential TTE; dynamic regimes | pCR/MPR, EFS, OS, surgical delay, QoL | Prospective strategy trial or MDT validation | Assuming pCR automatically implies long-term benefit |
| RT-immunotherapy | Balance local control and pneumonitis | ICI alone vs concurrent/sequential RT/SABR plus ICI | TTE, MSM, joint efficacy-toxicity utility | Local control, PFS/OS, pneumonitis, discontinuation | Dose-timing standardization and federated imaging validation | Immortal-time bias from completed combination therapy |
| irAE management | Continue, interrupt, de-escalate or rechallenge? | Continuation vs interruption; rechallenge vs switch | Dynamic regimes; safe RL; net-benefit models | Severe irAEs, hospitalization, OS/PFS, recurrence, QoL | Validated toxicity extraction and patient-preference weights | Using toxicity risk alone as a decision rule |
| Discontinuation/rechallenge | Can durable responders stop safely? | Continue vs planned discontinuation vs monitoring or rechallenge | Landmark TTE; sequential trials; CATE | Relapse, OS, PFS2, recurrent irAEs, treatment burden | Long-term follow-up and explicit response-state time zero | Equating relapse prediction with effect of continuing therapy |
| Weak-evidence populations | How should trial-ineligible patients be treated? | Standard vs modified or supportive-first strategies | Pragmatic TTE; transportability/fairness analysis | OS/PFS, hospitalization, toxicity, patient-reported outcomes | Multicentre validation and subgroup calibration | Interpreting access-to-care differences as biology |

These use cases illustrate strategy-level questions in which risk prediction alone is insufficient. Each scenario should be translated into executable alternatives, then evaluated through target trial emulation, effect-modification analysis, dynamic-regime modelling or reinforcement learning as appropriate. Clinical translation requires external validation, toxicity-aware net-benefit assessment and multidisciplinary review
